# Design and Analysis of Receiver Coils with Multiple In-Series Windings for Inductive Eddy Current Angle Position Sensors Based on Coupling of Coils on Printed Circuit Boards

**DOI:** 10.3390/s24154880

**Published:** 2024-07-27

**Authors:** Stefan Kuntz, Daniel Gerber, Gerald Gerlach, Sina Fella

**Affiliations:** 1 Robert Bosch GmbH, 74232 Abstatt, Germany; 2 Faculty of Electrical and Computer Engineering, Dresden University of Technology, 01069 Dresden, Germany

**Keywords:** inductive sensors, rotary position sensors, angular position sensors, encoders, coil design, PCB coils, angular error, inductive multiwinding design

## Abstract

We present a method for improving the amplitude and angular error of inductive position sensors, by advancing the design of receiver coil systems with multiple windings on two layers of a printed circuit board. Multiple phase-shifted windings are connected in series, resulting in an increased amplitude of the induced voltage while decreasing the angular error of the sensor. The amplitude increase for a specific number of windings can be predicted in closed form. Windings are placed electrically in series by means of a differential connection structure, without adversely affecting the signal quality while requiring a minimal amount of space in the layout. Further, we introduce a receiver coil centerline function which specifically enables dense, space-constrained designs. It allows for maximization of the number of possible coil windings while minimizing the impact on angular error. This compromise can be fine-tuned freely with a shape parameter. The application to a typical rotary encoder design for motor control applications with five periods is presented as an example and analyzed in detail by 3D finite-element simulation of 18 different variants, varying both the number of windings and the type of centerline functions. The best peak-to-peak angular error achieved in the examples is smaller than 0.1° electrically (0.02° mechanically, periodicity 5) under nominal tolerance conditions, in addition to an amplitude increase of more than 170% compared to a conventional design which exhibits more than twice the angular error. Amplitude gains of more than 270% are achieved at the expense of increased angular error.

## 1. Introduction

Angle position sensors are crucial to many systems in industrial and automotive applications, such as motor control [1]. A variety of measurement principles exist, such as magnetic, optical, capacitive, and inductive sensors [2].

**Figure 1:**
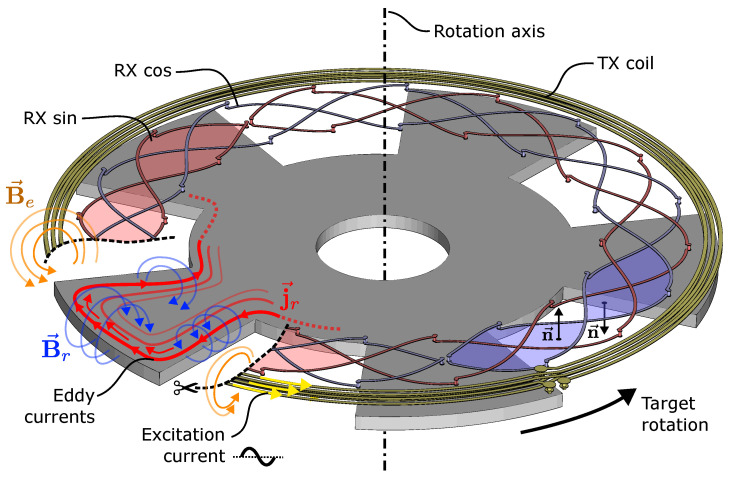
Overview of the operating principle of a rotary inductive position sensor. A ∼4 MHz AC current through the transmitter coil (TX) generates a magnetic excitation field $B_{e}$ which induces eddy currents $j_{r}$ in an electrically conductive target material, which in turn generates an opposing reaction field $B_{r}$ that induces a voltage in the receiver coils (RX). Due to the shape of the target and the spatial phase shift between the two receiver coils, the target angle can be measured by calculating the angle of the demodulated receiver coil voltages. The differential nature of the RX coils suppresses direct coupling of TX coil into the RX coils due to the inverted surface normals n of the RX coil area segments.

There are several measurement principles which might qualify as inductive sensing, such as simple binary proximity switches [3], non-destructive testing technology [4], displacement sensors [5], and possibly linear variable differential transformers (LVDTs) [6], as well as lresolvers [7,8]. While the latter two principles primarily rely on magnetic permeability effects, other inductive sensors rely on induced eddy currents—also known as Foucault’s currents—as the main operating principle. Celikel et al. consider inductive angle position sensors to be a subset of resolvers in their review [9]; however, it is important to note that the measurement principle differs (Figure 1).

Generally, angle position sensors are characterized by the measurement of at least two signals which encode an angle signal, which is usually reconstructed by application of the arctangent function to determine the position more robustly [2]. These signals are often referred to as being in quadrature as sine and cosine channels. Variants utilizing more than two signals are known, e.g., three-phase designs, which utilize a transformation (e.g., the Clarke transform) to reduce to the two-phase case [10]. Note that angular position sensing is applicable to both rotary and linear position sensors, since an electrical angle is measured in both cases—in the linear case, the angle merely corresponds to a linear movement along an axis.

However, even for inductive sensors based on eddy currents, a distinction must be made regarding the exact sensing principle which is used to measure these signals. There are two main inductive principles which are often used, which are sometimes referred to as *individual coil* and *coupled coil* principles.

In the individual coil principle, at least one coil forms an LC-oscillator circuit, which oscillates at a nominal operating frequency. The position of the target is measured by a change in the resonance frequency due to the eddy currents induced in the target. There are variants for distance measurement which essentially use a single coil to achieve this, while more advanced variants of this sensor type, such as, e.g., that presented by Vogel et al., use multiple coils/oscillators for *ratiometric measurement* [11,12]. Individual coil angle sensors use multiple coils and oscillators which change their *inductance* in quadrature for angle calculation [13,14].

On the other hand, *coupled coil* sensors differ fundamentally. They consist of a *transmitter* coil (TX) and *receiver* (RX) coils, sometimes also referred to as *excitation* and *pickup* coils. An electric current flowing through the transmitter coil creates a magnetic field at a frequency of typically several megahertz, which induces eddy currents in a coupling element (target). The magnetic field resulting from these eddy currents varies the effective coupling between transmitter and receiver coils, depending on the position of the target (Figure 1). Since more challenging synchronous demodulation is required to process the induced signals in the receiver coils, the demodulation circuitry is typically contained in an application-specific integrated circuit (ASIC) [15,16,17]. Typically, the coils are constructed as traces on a printed circuit board (PCB), which is cost-effective due to a well-controlled manufacturing process and high resulting accuracy of the coil layout.

The fundamental coil design of such inductive position sensors has seen few real-world improvements since the first commercial introduction in the automotive industry in the early 2000s [18,19,20]. Further, scientific literature on the subject is sparse, since such sensors are typically designed and developed in proprietary industrial settings such as the automotive industry [21] and often published only as patents, if at all. Still, several authors [22,23,24,25] present coupled coil sensors based on receiver coils generated from a cosine function. Hoxha et al. present an optimized coil geometry starting from a cosine design [26]. Zhang et al. use spiral receiver coils [27,28], which, however, restricts the possible periodicity. Ye et al. use receiver coils based on a rectangular function [29], but do not provide a parameterization. A novel idea on the use of multiple windings in the receiver coil layout was previously presented in a patent application by Ausserlechner [30] with the intent to reduce the angular error of the sensor, which provided great potential for further research.

In the following, we improve upon the basic concept of multiple receiver coil windings outlined in [30] in several ways. First of all, a practical, space-efficient, and generally applicable connection structure to connect windings electrically in series is developed. Specifically, the structure does not require additional PCB layers and only a small amount of radial space. Furthermore, we provide alternative receiver coil centerline functions which can be used in case of intersections or PCB design rule issues in dense designs. By introduction of a continuous shape parameter, the tradeoff between angular error and amplitude loss in contrast to the minimum copper-to-copper distance in a design can be fine-tuned.

For the sake of brevity, we restrict the explanations to a *rotary* position sensor in the following. However, the concepts developed in this paper are also applicable to other types of inductive position sensors. A common and similar application is the development of linear inductive sensors that measure the movement of a target along a straight axis with an appropriate linear coil design, e.g., detailed in [31,32].

Many of the fundamental choices for the design of coils presented here, such as some centerline functions, via placements, etc., are not new and date back to at least the year 1999 [33]. However, we still include them here, since our contribution also lies in the comprehensive and extensible description of the design process with a particular focus on mathematical parameterization. The primary goal of this research is to enable new inductive sensing applications which are not possible with state-of-the-art concepts, such as small and highly integrated sensors embedded in actuators with strict angular error requirements and limited capabilities for digital compensations of such errors. Our work is structured into two main parts. We first focus on a generally applicable parameterization of receiver coil functions for rotary inductive position sensors and the transformation steps required to provide a functioning coil layout. We further analyze the effect of multiple phase-shifted windings on the amplitude and angular error when the windings are connected in series. Secondly, we develop a concept to efficiently connect windings in series in a space-constrained application, while keeping the same number of PCB layers without negatively impacting the signal quality.

## 2. Design of Receiver Coils

In theory, an inductive rotary position sensor can be built with a wide range of receiver coil designs. However, there are only a few choices that are useful in practice, which share common similarities. We therefore restrict the following definitions to a subset of applicable coil designs with high relevance, but with enough flexibility to cover a wide range of rotary position sensing applications. Our intent is to provide a parametric process which particularly enables the automated generation of such coil designs with software tools.

Notably, the described receiver coils are all *differential* in nature (Figure 1 and Figure 4). A homogeneous or circularly symmetric AC magnetic field along the z-axis perpendicular to the PCB induces approximately zero voltage in the coils. Further, the magnetic field of the circular TX coil also induces approximately zero voltage. Only the presence of the target with its *wings* (Figure 1 and Figure 10) breaks the differentiality and leads to an induced voltage.

All practically useful sensor designs consist of more than one *phase* to enable calculation of an angle with the arctangent function. We denote the number of phases by *m*; therefore, m≥2. In our concrete examples, we use m=2, since this is the most common choice for ASICs on the market. However, all parameterizations allow m>2 without issue.

In safety-critical or high-availability applications, *redundant* coil systems may be used which are connected to separate ASICs. If the redundancy is achieved by duplication of the coil system on other PCB layers where it has no significant effect on the layout of the individual coil systems, we do not consider it further in the following. However, if the redundancy is achieved by re-using the same via positions or PCB layers, therefore requiring a phase-shifted redundant system, the number of *systems* can be considered by increasing the number of phases *m* accordingly. It therefore defines the total *number of receiver coils* of the sensor in this case. The distinction between phases and coils is dependent solely on signal processing.

A *coil* is a continuous trace on the printed circuit board which is closed, or at least almost closed, and may traverse several PCB layers with *vias*. A single coil may consist of a number of windings $n_{w}$ if they are connected in series electrically.

Further, we denote by *p* the *periodicity* of the sensor, which implies *p*-fold symmetry of the target and approximate *p*-fold symmetry of the receiving coils in the considered examples, as long as connection structures are not considered. The mechanical rotation angle of the target is denoted by ϕ, while an angle in polar coordinates which does not correspond to a mechanical rotation is denoted by φ.

### 2.1. Coil Centerline Functions

**Figure 2:**
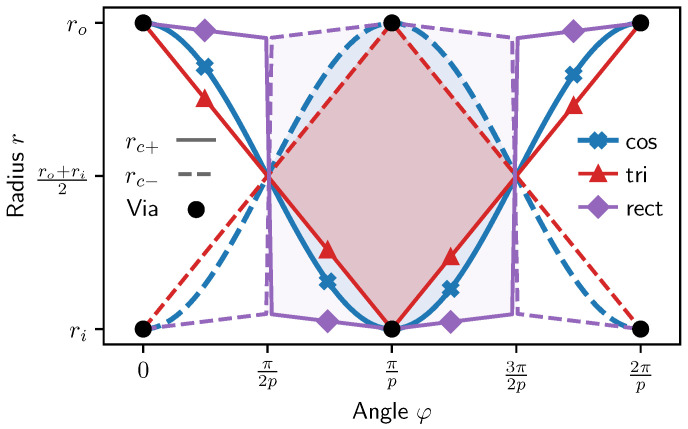
Receiver coil centerline function examples in polar coordinates for three common contours: cosine (cos), triangular (tri), and modified rectangular (rect) functions. The surface area (partially shaded in color) spanned by a receiver coil is defined by the pair $r_{c+}\left(\varphi \right)$ (solid lines) and its symmetric counterpart $r_{c-}\left(\varphi \right)$ in opposite winding direction (dashed lines). Black dots indicate the location for vias to traverse the layers (not shown) of the PCB to prevent the intersection of copper traces.

The following section is focused on the generation of the fundamental receiver coil geometry and is generally applicable to rotary inductive angle position sensors while not being limited to designs with multiple windings in series. In the case of a rotary sensor, the path of the receiver coil trace on the PCB is best defined in polar coordinates as r(φ), yielding a radius *r* of the trace centerline depending on the polar angle φ around the center of the coil system (Figure 2). For manufacturing, the mathematical centerline description is *extruded* with the trace width and copper layer thickness to obtain the description of the actual conductor volume.

In order to decouple the elementary *shape* of the receiver coil from other sensor parameters, e.g., inner/outer radius and periodicity, we define a *unit centerline function* $r_{c}\left(\tilde{\varphi }\right)$. It defines the normalized radius *r* for a given periodicity-normalized angle $\tilde{\varphi }$. Therefore, $r_{c}\left(\tilde{\varphi }\right)$ is independent of the concrete sensor design parameters and only defines the intrinsic *shape* of the centerline function. The unit centerline function can then be converted to the sensor-specific centerline functions $r_{c+}$ and $r_{c-}$ taking into account the inner/outer radius and coil periodicity of the design. A similar parameterization is used by Lugani et al. [34], which, however, requires a periodic unit centerline function, which is disadvantageous, especially when constructing $r_{c}\left(\tilde{\varphi }\right)$ from discrete points (e.g., Section 2.1.3).

Starting with an arbitrary basic unit receiver coil centerline function

(1) $$r_{c}\left(\tilde{\varphi }\right)\in \left[-1,1\right]$$

defined over the interval $\tilde{\varphi }\in \left[0,2\pi \right)$, the sensor periodicity is considered by converting the angle φ in polar coordinates to the normalized angle $\tilde{\varphi }$ used in the $r_{c}\left(\tilde{\varphi }\right)$ definition:

(2) $$\tilde{\varphi }=p\varphi \;\mathrm{mod}\;2\pi =p\varphi -2\pi \left\lfloor \frac{p\varphi }{2\pi }\right\rfloor \;,$$

where *p* is the sensor periodicity, mod is the modulo operation [35], and $\left\lfloor \ldots \right\rfloor$ is the floor function. Note that in case of negative numbers, the modulo operation commonly available in programming languages is implementation-dependent [36]. Therefore, care should be taken with negative angle values when using a modulo-based implementation.

A receiver coil consists of differential segments which are constructed by combining a *positive* and *negative* centerline function. The positive centerline function for a specific outer radius $r_{o}$ and inner radius $r_{i}$ is obtained by

(3) rc+(φ)=−arc(φ˜)+o

and the negative (inverse) centerline function by

(4) $$\begin{array}{cc} r_{c-}\left(\varphi \right) & =-a\;r_{c}\left(\tilde{\varphi }\right)+o \end{array}$$

where the amplitude *a* and radial offset *o* of the contour are given by

(5) $$\begin{array}{cc} a & =\frac{r_{o}-r_{i}}{2}, \end{array}$$

(6) $$\begin{array}{cc} o & =\frac{r_{o}+r_{i}}{2}\;. \end{array}$$

Possible receiver coil designs are therefore restricted to those with intrinsic symmetry of positive and negative centerlines around $r_{m}=\frac{r_{i}+r_{o}}{2}$ by definition. However, the symmetry of positive and negative *area segments* spanned by the windings between $r_{c+}$ and $r_{c-}$ must be ensured by the definition of $r_{c}\left(\tilde{\varphi }\right)$ for a full-differential design. It is only given if

(7) $$\begin{array}{cc} \int_{0}^{2\pi }r_{c}\left(\tilde{\varphi }\right)\varphi & =0\;, \end{array}$$

which can easily be ensured by imposing, e.g.,

(8) $$r_{c}\left(\tilde{\varphi }+\pi \right)\overset{!}{=}-r_{c}\left(\tilde{\varphi }\right)$$

for $\tilde{\varphi }\in \left[0,\pi \right)$.

From Equation (7), it also follows that the induced voltage in the receiver coil is zero in a homogeneous magnetic field in the z-direction due to the differential construction of the coil:

(9) $$\begin{array}{cc} \int_{0}^{2\pi }\int_{r_{c-}\left(\varphi \right)}^{r_{c+}\left(\varphi \right)}B_{z}rdrd\varphi & =0\;. \end{array}$$

This property is fulfilled by all coils constructed with the presented $r_{c}\left(\varphi \right)$ formalism under the assumption that they are perfectly *flat* with no spatial extent in the z-direction (i.e., zero distance between PCB layers). For a complete parameterization of the coil surface for integration, including the small extension in z-direction, see [25]. In practice, this is not possible, because the coil traces would self-intersect, leading to a short circuit. Therefore, the traces have to be distributed onto at least two PCB layers, as shown in the following.

The actual coil centerline on the PCB is obtained by transformation from polar coordinates r(φ) to Cartesian coordinates:

(10) $$\begin{array}{cc} x_{+} & =\left[\begin{array}{c} r_{c+}\left(\varphi \right)\cos\varphi \\ r_{c+}\left(\varphi \right)\sin\varphi \end{array}\right] \end{array}$$

(11) $$\begin{array}{cc} x_{-} & =\left[\begin{array}{c} r_{c-}\left(\varphi \right)\cos\varphi \\ r_{c-}\left(\varphi \right)\sin\varphi \end{array}\right] \end{array}$$

applied to $r_{c+}$ and $r_{c-}$. Sweeping 0≤φ<2π yields two separate closed loops from $x_{+}$ and $x_{-}$ based on the fundamental shape of the receiver coil centerline function.

The above definition still contains two major issues. First, the loops for $x_{+}$ and $x_{-}$ intersect, which is solved by utilizing several layers of the PCB, which are traversed with vias. Further, the loops are *closed* and therefore electrically shorted themselves. For the coupled coil inductive measurement principle, the receiver coils are connected to an ASIC and terminated with high impedance. The induced voltage is measured with minimal load on the coil at the operating frequency. Shorting receiver coils creates an additional magnetic field due to currents induced in the coil itself, which disturbs the measurement and is not desired. In order to form a differential structure, the loops also have to be connected together, instead of forming two independent segments. Therefore, additional modifications are required in order to obtain the proper coil geometry.

Assuming that the centerline function $r_{c}\left(\tilde{\varphi }\right)$ has an approximate maximum at $\tilde{\varphi }=0$ and approximate minimum at $\tilde{\varphi }=\pi$, i.e., is *cosine-like*, vias are then placed on the outer and inner radius at every angle increment

(12) $$\varphi_{k}=\frac{k\pi }{p}$$

for k∈{0,1,…,2p−1}. At every via, the PCB layer of the centerline trace is swapped to avoid intersection.

#### 2.1.1. Cosine Centerline Function

In order to obtain harmonic sine/cosine output signals from an inductive position sensor when rotating the target, a sinusoidal coil centerline function is the natural choice. In the following, we refer to this type as a *cosine* centerline function.

It can be obtained by simply letting

(13) $$r_{c}\left(\tilde{\varphi }\right)=\cos\left(\tilde{\varphi }\right)\;.$$

Generally, a cosine centerline function leads to the lowest angular error compared to other variants—with the exception of complex coil geometries optimized for a specific design. While it seems intuitive, this can also be shown by mathematical analysis of the coil signals if a number of simplifying assumptions are made. In order to understand the details of angular error, we refer to [37]. Notably, the angular error is zero if higher-order harmonics in the sensor signals vanish, and only the *pure p*-th-order harmonic remains in both the sine and the cosine channels—given that they are offset-free, amplitude-matched, and orthogonal.

Specifically, we assume a concentric circular TX coil structure centered around (0,0) and an arbitrarily shaped target with *p*-fold symmetry centered and rotating around (0,0), i.e., ideal mechanical alignment. We consider a single receiver coil generated with the formalism described in Section 2.1, with a centerline function r(φ). The receiver coil is assumed to be flat with no spatial extent in the *z*-direction, and the traces are assumed to be thin filaments. The receiver coil surface is consequently reduced to a surface in the xy-plane. Note that with regard to the induced voltage, a rotation of the target is then equivalent to a rotation of the receiver coils.

These simplifying assumptions are adequate for the vast majority of inductive sensor designs, because the trace width (∼125 μm), copper layer height on PCB (∼35 μm), and distance between PCB layers (∼200 μm) are all small compared to the diameter of the coil area (usually d≳20 mm).

Under these assumptions, it can be shown that *any radially homogeneous*, *p*-periodic magnetic field in the RX coil plane $B_{z}\left(\varphi \right)$ from the target eddy currents yields pure harmonic induced voltages of order *p*. Therefore, the resulting angular error from any RX coil arrangement is *zero*, if the centerline function consists only of the fundamental harmonic. Under these conditions, the cosine centerline function is angular-error-optimal.

Faraday’s law states that the EMF ϵ in the receiver coil can be expressed as the integral over the surface *S* spanned by the RX coil. Since there is a phase shift between the transmitter voltage and the resulting eddy current fields depending on the inductive and ohmic effects in the system, the integration would best be performed over a complex magnetic field $B\in C^{3}$. However, we model the demodulation of the ASIC by taking the real part when assuming the TX voltage as the phase reference for synchronous demodulation. If we assume no electrical load on the RX coil, this is equal to the measured induced voltage, dependent on the target rotation angle ϕ:

(14) $$\begin{array}{cc} V_{\mathrm{RX}}\left(\phi \right) & =-\frac{\partial }{\partial t}\underset{S}{\;\int \int \;}B\;dS\;. \end{array}$$

In polar coordinates, assuming vanishing extent of the RX coil in the z-direction and harmonic excitation, we obtain

(15) $$V_{\mathrm{RX}}\left(\phi \right)=j\omega \int_{0}^{2\pi }\int_{r_{c-}\left(\varphi \right)}^{r_{c+}\left(\varphi \right)}B_{z}\;r\;dr\;d\varphi \;.$$

Due to assumed radial homogeneity of *B_z_*,

(16) $$V_{\mathrm{RX}}\left(\phi \right)=j\omega \int_{0}^{2\pi }B_{z}\int_{r_{c-}\left(\varphi \right)}^{r_{c+}\left(\varphi \right)}r\;dr\;d\varphi$$

(17) $$\;\;\;\;\;\;\;\;\;\;\;\;\;\;\;\;\;\;\;\;\;\;\;\;\;\;\;\;\;\;\;\;\;=\frac{1}{2}j\omega \left(\int_{0}^{2\pi }B_{z}r_{c+}^{2}\;d\varphi -\int_{0}^{2\pi }B_{z}r_{c-}^{2}\;d\varphi \right)\;.$$

Let $r_{c}(\tilde{\varphi })=\cos(\tilde{\varphi })$, then

(18) $$V_{\mathrm{RX}}\left(\phi \right)=2j\omega \;ao\int_{0}^{2\pi }B_{z}\cos\left(p\varphi \right)\;d\varphi$$

with *a* and *o* from Equations (5) and (6). Of course, $B_{z}$ depends on the target rotation ϕ and is evaluated at a point (r,φ) in polar coordinates. Under our assumptions, $B_{z}$ does not depend on *r* due to assumed radial uniformity and ϕ simply offsets φ due to ideal alignment, resulting in $B_{z}\left(\varphi -\phi \right)$. It is apparent that only a single harmonic of the magnetic field provides a contribution to the value of the integral. Continuing Equation (18),

(19) $$\begin{array}{cc} V_{\mathrm{RX}}\left(\phi \right) & =\frac{1}{2}j\omega \;\left(r_{o}^{2}-r_{i}^{2}\right)\int_{0}^{2\pi }B_{z}\left(\varphi -\phi \right)\cos\left(p\varphi \right)\;d\varphi \;. \end{array}$$

Since *B_z_* is *p*-periodic and harmonics of different frequency are orthogonal when integrating *φ* over the interval [0, 2*π*),

(20) $$V_{\mathrm{RX}}\left(\phi \right)\propto \frac{1}{2}j\omega \;\left(r_{o}^{2}-r_{i}^{2}\right)\cos\left(p\phi \right)\;$$

(21) $$\begin{array}{cc} \; & \propto \cos(p\phi )\;. \end{array}$$

Therefore, only the *p*-th-order harmonic of $B_{z}$ remains, which results in zero angular error under the assumptions made. Note that we did not assume any behavior of $B_{z}\left(\varphi \right)$ in angular direction except *p*-periodicity. The cosine centerline function acts like an ideal *filter* against all other harmonics. Other receiver coil centerline functions contain higher-order harmonics in $r_{c}\left(\tilde{\varphi }\right)$ itself, which introduce higher-order harmonics in the induced voltage, thus resulting in an increased angular error. Nonetheless, these choices can be useful, as shown in the following for selected centerline functions.

#### 2.1.2. Triangle Centerline Function

One of the easiest centerline functions that can be constructed is the *triangle* shape, also seen in [18]. As we will show later, the triangle function on its own is not particularly useful due to high angular error and low induced voltage amplitude, but it serves as a basis for more advanced receiver coil designs.

The normalized triangle centerline function is given by

(22) rc(φ˜)=−2πφ˜+1for0≤φ˜<π−2πφ˜−3forπ≤φ˜<2π

without dependence on additional parameters.

#### 2.1.3. Modified Rectangle Centerline Function

In order to maximize the differential surface area of the receiver coil, and therefore the induced signal amplitude, a *rectangular* centerline function can be chosen. However, a true rectangular shape is not possible on printed circuit boards because of intersections of traces with the required vias of other windings or phases. Therefore, it has to be modified in order to avoid these intersections, resulting in a hexagon-like structure in polar coordinates (Figure 2) which we further refer to as the *modified rectangle* centerline function.

It is obtained by:

(23) $$\begin{array}{cc} r_{c}\left(\tilde{\varphi }\right) & =\left\{\begin{array}{cc} -\frac{2\Delta r_{o}}{\pi }\tilde{\varphi }+1 & \mathrm{for}\;0\leq \tilde{\varphi }<\frac{\pi }{2}\\ -\frac{2\Delta r_{i}}{\pi }\tilde{\varphi }+2\Delta r_{i}-1 & \mathrm{for}\;\frac{\pi }{2}\leq \tilde{\varphi }<\pi \\ \frac{2\Delta r_{i}}{\pi }\tilde{\varphi }-2\Delta r_{i}-1 & \mathrm{for}\;\pi \leq \tilde{\varphi }<\frac{3\pi }{2}\\ \frac{2\Delta r_{o}}{\pi }\tilde{\varphi }-4\Delta r_{o}+1 & \mathrm{for}\;\frac{3\pi }{2}\leq \tilde{\varphi }<2\pi \end{array}\right. \end{array}$$

which is discontinuous at $\tilde{\varphi }=\frac{\pi }{2}$ and $\tilde{\varphi }=\frac{3\pi }{2}$. However, the discontinuities in polar coordinates are not relevant when discretizing φ numerically, as done when generating an actual coil design. It is treated as a simple radial connection between the corresponding endpoints. $r_{c}\left(\tilde{\varphi }\right)$ of the modified rectangular centerline function depends on two additional parameters $\Delta r_{i}$ and $\Delta r_{o}$, which are the normalized radial lengths that define the slope of the *hat* segments on inner and outer radius up to the discontinuity, and therefore how “pointy” the top and bottom of the function are. Both are given in terms of normalized radii, equivalent to an absolute delta if the amplitude of the contour were equal to 1 ($r_{o}=1$, $r_{i}=-1$).

#### 2.1.4. Modified Cosine Centerline Function

**Figure 3:**
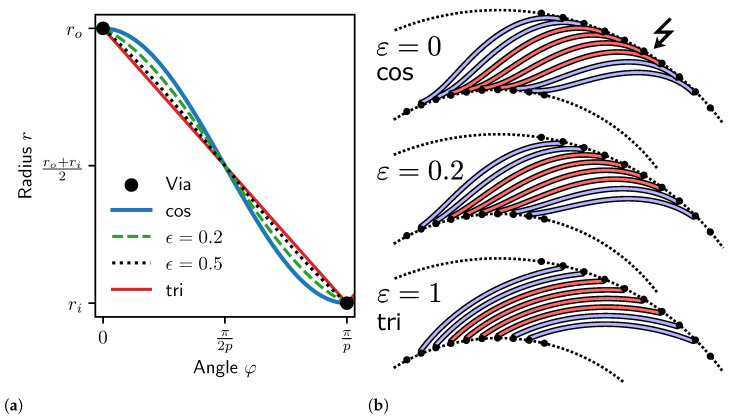
Receiver coil centerline functions $r_{c+}$ for modified sinusoidal contours (ϵ=0.2 and ϵ=0.5) compared to cosine (cos, ϵ=0) and triangular (tri, ϵ=1) in polar coordinates (**a**) and transformed to Cartesian coordinates for $n_{w}=4$ (**b**). The first half of a full period is shown. ϵ>0 increases the slope of the centerline function at the extrema, allowing for closer via placement of neighboring windings without intersection even in dense designs.

In space-constrained designs, such as those with many in-series windings, intersections between vias and adjacent traces can occur due to an insufficient slope of the centerline function close to the maxima (Figure 3b). For example, in case of the cosine centerline function, the slope given by the first-order derivative is zero at the maxima and only slowly increases in the vicinity (Figure 3a).

The previously introduced triangle centerline function solves this issue due to the large slope at the maximum but is undesirable due to excessive angular error. Therefore, we introduce a new centerline function with an additional shape parameter ϵ, which enables continuous *morphing* between a cosine and triangle function. This has the advantage that the slope of the contour at the vias can be gradually increased, so that more PCB traces can fit into a design, given the same PCB manufacturing constraints with regard to the distance of two adjacent copper areas. At the same time, the angular error is kept as low as possible by not fully utilizing the pure triangular centerline function. instead keeping a more cosine-like shape. The parameter ϵ can be fine-tuned to enable an optimal design under specific constraints for the sensor geometry.

It is particularly useful in coil arrangements with many windings ($n_{w}>1$). The effect of the periodicity *p* on the copper distance constraints is typically less critical than the increase in the number of windings $n_{w}$ or number of phases *m*.

The *modified cosine* centerline function is obtained by:

(24) $$\begin{array}{cc} r_{c}\left(\tilde{\varphi }\right) & =\left\{\begin{array}{cc} \frac{1}{\cos\left(\frac{\pi }{2}\epsilon \right)}\cos\left(\left(1-\epsilon \right)\tilde{\varphi }+\frac{\pi }{2}\epsilon \right) & \mathrm{for}\;0\leq \tilde{\varphi }<\pi \;,\\ \frac{1}{\cos\left(\frac{\pi }{2}\epsilon \right)}\cos\left(\left(1-\epsilon \right)\left(2\pi -\tilde{\varphi }\right)+\frac{\pi }{2}\epsilon \right) & \mathrm{for}\;\pi \leq \tilde{\varphi }<2\pi \;, \end{array}\right. \end{array}$$

where ϵ∈[0,1). For ϵ=0 one obtains a cosine function, for ϵ=1, a triangle function. Note that Equation (24) is mathematically undefined for ϵ=1, but approaches a triangular centerline function in the limit ϵ→1. Numerically, this case has to be implemented by additional case discrimination if ϵ≈1, replacing $r_{c}\left(\tilde{\varphi }\right)$ with a triangular function in this case. The floating point resolution of the actual implementation should be considered for this case distinction.

Equation (24) is based on a regular cosine, but a fraction of a period is stretched to cover the full angle range. One half of a period of the cosine function is restricted to the interval $\left\{\frac{\pi }{2}\epsilon ,\frac{\pi }{2}\left(2-\epsilon \right)\right\}$, where 0≤ϵ<1. The result is stretched again to the interval [0,π]. The second segment $\pi \leq \tilde{\varphi }<2\pi$ is created by simply mirroring the segment $\;0\leq \tilde{\varphi }<\pi$ around $\tilde{\varphi }=\pi$. The amplitude of these sections must be modified for proper normalization to match the intended amplitude of 1. It also becomes apparent that ϵ=1 is essentially stretching a point to a line, thus the need for case distinction.

The modified cosine is highly relevant for real-world applications, e.g., our example designs $n_{w}=4$ and $n_{w}=5$ are so dense that the ordinary cosine centerline function cannot be used without intersections. Such designs are made possible by the proposed modified cosine with ϵ>0, while keeping the angular error acceptably small. Note that a similar effect with regard to a higher via-to-trace distance can also be obtained by reducing the amplitude of the receiver coil function and adding radial traces to the vias (unchanged at the same position). However, this comes at the cost of higher loss of amplitude due to the shrinking of the coil. Both methods can be combined.

### 2.2. Additional Required Modifications

**Figure 4:**
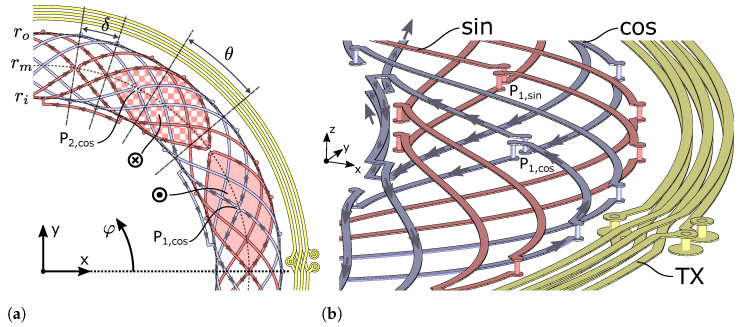
Receiver coils of a sensor design with periodicity p=5, two phases (m=2, “sin”, “cos”), and two in-series windings per phase ($n_{w}=2$) spanning two layers of the PCB in z-direction. RX coil phases are phase-shifted relative to each other by 90° electrically, equivalent to θ=90°/p=18° mechanical rotation. Two windings are connected in series with a differential connection segment on both layers (note opposing directions on upper and lower layer). Shaded areas indicate a part of the surface area spanned by the receiver coil winding around the location of the reversal point ($P_{1,\sin}$) of the winding, with their surface normal in positive (filled) and negative (checkered) z-direction. The transmitter coil (TX) consists of 3 windings on two layers. (**a**) Top view. (**b**) Detailed perspective.

Based on the formalism in Equations (1)–(10), two separate *closed* receiver coil loops are obtained, one for $r_{c+}$ and one for $r_{c-}$. In order to facilitate a *differential* measurement, the two loops are combined to form differential segments with alternating surface normal direction (Figure 4b). There are several possible contour modifications to achieve this. In the simplest case, a *reversal point* is placed for each coil, cutting the contour loops in a common location and connecting $r_{c+}$ to $r_{c-}$ on each of the two ends of the cut with a via (Figure 4a). The result is a single closed loop, comprising the whole of $r_{c+}$ and $r_{c+-}$. When traversing the loop, the general direction of the contour changes from clockwise to counter-clockwise or vice versa at the reversal point, hence its name.

In order to measure the induced voltage in the coil and in order to avoid a short-circuit, which would result in current flow in the receiver coil, the loop is cut at an arbitrary position to create the open coil terminals. In the actual application, the coil is then connected to the inductive ASIC at the terminals. In finite element simulation, a *coil port* is placed at this location.

The above is valid for a single winding of a single phase of the coil system. The same process is repeated for all phases of the coil system, but additional complexity is introduced in the case of multiple windings, which require special care in the connection of the windings.

The coil geometry of the additional phases is obtained by successive duplication and mechanical rotation by

(25) $$\theta =\frac{\pi }{mp}\;.$$

For example, a three-phase m=3 system with periodicity p=1 exhibits a mechanical phase shift of θ=60° between coils, while a two-phase system with p=5 has its coils shifted by θ=18° to each other (Figure 4).

**Figure 5:**
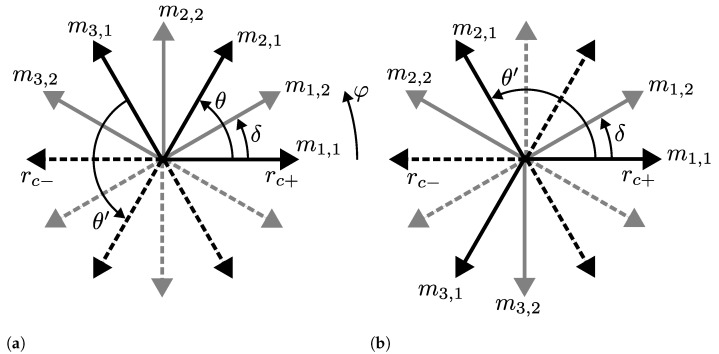
Equivalence of the phase shift (Equation (25)) between coil phases of a three-phase m=3 design with two windings $n_{w}=2$ (black, gray) spaced with uniform δ, assuming p=1 to avoid distinction between electrical and mechanical angles. An angle of θ=60° (**a**) and $\theta^{\prime }=120{}^{\circ}$ (**b**) between phases leads to the same coil layout geometrically, except for the polarity definition of every other coil by swapping $r_{c+}$ (solid lines) and $r_{c-}$ (dashed).

The attentive reader might notice that Equation (25) leads to θ=60° between phases in a three-phase system, rather than the well-known phase shift of 120°. We avoid a case distinction between odd and even numbers of phases in Equation (25) to rectify this because the obtained coil layout is geometrically identical in both cases and only differs in the definition of coil polarity by swapping $r_{c+}$ and $r_{c-}$ (Figure 5). This applies to any coil layout with an *odd* number of phases. The polarity of coils requires careful attention in a real-world application anyway, in order to ensure the intended rotation direction of the angle signal.

### 2.3. Multiple Phase-Shifted Windings in Series

Often, one of the main challenges when designing an inductive sensor is reaching the minimum amplitude required by the IC specification, especially when dealing with large airgaps between the target and the coils on the PCB. By utilizing more than one winding of the receiver coils on the PCB, the amplitude of the induced voltage can be increased. However, connecting the windings in series without intersections and without adversely affecting the angular error is not trivial. The easiest solution is the utilization of more PCB layers, which increases the PCB cost. In order to avoid this, the additional receiver coil windings are created by duplication of the single-winding receiver coil geometry and subsequent phase-shifting to enable placement on the existing layers of the PCB. The separate loops then must be connected in series electrically.

In principle, the phase shift angle δ between two adjacent windings of the same phase can be chosen arbitrarily as long as it does not lead to intersections of the traces and vias on the PCB. In order to achieve a maximum density with as many windings as possible, an equidistant uniform spacing of the windings of all phases is desirable. The mechanical angle between two windings for this case is obtained by

(26) $$\delta_{\mathrm{uniform}}=\frac{\pi }{m\;n_{w}\;p}$$

which is also the angle between two adjacent vias (Figure 3a). We define δ≥0 for convenience. The mechanical rotation angle δ of a winding is related to the *electrical* phase shift $\delta_{p}$ between windings by

(27) $$\delta =\frac{\delta_{p}}{p}\;,$$

i.e., for higher sensor periodicities, the mechanical rotation angle is smaller than the resulting electrical phase shift. We take $\delta_{p}$ as the given parameter in the following sections, since it makes equations and figures independent of the actual sensor periodicity *p*. However, it is important to keep in mind that $\delta_{p}$ is merely a consequence of the *mechanical* phase shift (i.e., rotation during duplication) between the coil windings by δ. We further restrict our analyses to constant δ between any two adjacent windings of a coil for simplicity.

Equidistant spacing imposed by Equation (26) is not a necessity, but in dense designs with many windings, it presents the optimum with regard to maximization of copper-to-copper distances. In less dense designs where this is not critical, δ can be adjusted to either minimize the angular error or to maximize the induced voltage amplitude as long as PCB design rule constraints are fulfilled.

Since an influence of the in-series connection between windings on the angular error should be avoided, the connection structure is designed with two overlapping *differential* segments with opposite winding direction, so that their effect on the induced voltage effectively cancels out. Our proposed structure only requires minimal space on the inner or outer radius of the coil; therefore, the mechanical constraints are not affected in most cases.

A connection structure is created by replacing two vias between the two receiver coil layers with a trace on the same layers, which connects two receiver coil loops $r_{c+}$ and $r_{c-}$ of two windings (Figure 4). It is also possible to replace reversal points with such connection structures. In this case, the loops of a single winding are connected. Removing the necessity of a discrete reversal point in the middle of the coil can be beneficial in high-density applications.

Note that there is a large number of possibilities to place such connection structures, but only a small fraction of them produce a valid design. Solutions which produce valid designs are not necessarily unique; there can be many different connection patterns which result in a valid in-series connection of the coil windings. Some variants are beneficial, e.g., when the number of nested connections is reduced, which requires less space in radial direction (compare, e.g., Figures 8 and 9c).

### 2.4. Effect on the Induced Voltage Amplitude

**Figure 6:**
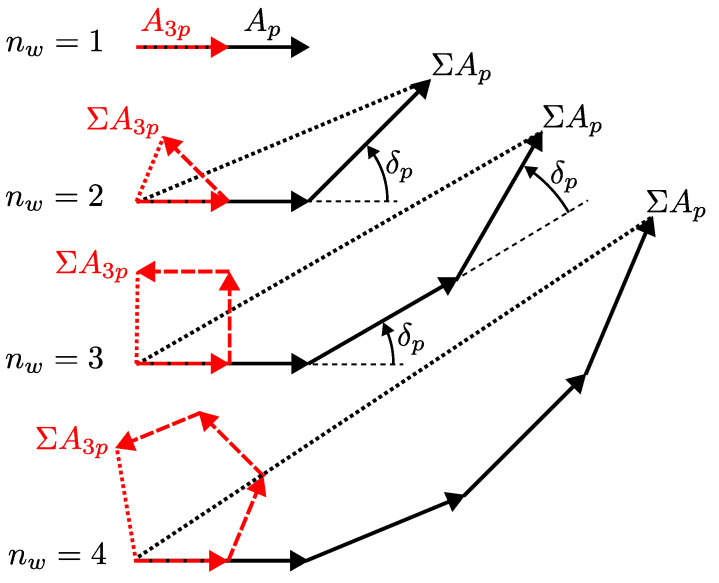
Superposition of winding voltages of the main harmonic with amplitude $A_{p}$ (black) and the 3rd harmonic with amplitude $A_{3p}$ (red) for the case of uniform winding phase shift (Table 1) of a two-phase design (m=2) and different number of windings $n_{w}$. The principle of constructive interference of the main harmonic with simultaneous suppression of higher-order harmonics is apparent.

Due to the in-series connection of two or more windings, the induced voltage in a coil is expected to increase. However, the increase is not directly proportional to the number of windings $n_{w}$ because of the phase shift required to establish the in-series connection without intersection. Since the receiver coil geometry has small planar extent and the coils are typically terminated with a high impedance, there is no significant increase in eddy currents in the receiver coil geometry due to the increased number of windings. Therefore, by representing the induced voltages in the individual windings as phasors which rotate with the target rotation except for a factor *p*, the effective induced voltage in the entire coil can be calculated by vector addition (Figure 6).

The effective amplitude $\Sigma A_{p}$ obtained by multiple windings in series can be calculated exactly in closed form if the assumptions listed in the following are fulfilled:

(28) $$\Sigma A_{p}=A_{p}\sqrt{\frac{\cos(\delta_{p}n_{w})-1}{\cos(\delta_{p})-1}}$$

where $A_{p}$ is the amplitude of the induced voltage harmonic of order *p* in a single winding, $\delta_{p}$ the electrical phase shift between consecutive windings of a coil, and $n_{w}$ the number of windings in series.

If no higher-order harmonics are present in the induced voltage signals, i.e., the angular error is zero when rotating the target, Equation (28) is directly applicable. If higher-order harmonics are present, the effective amplitude can be calculated for every harmonic order separately and Equation (28) is valid for the main harmonic, i.e., the mechanical order *p* which is the 1st electrical harmonic. For higher-order harmonics, the phase shift angle scales with the harmonic order, e.g., $\delta_{3p}=3\delta_{p}$ in Equation (28). Further, Equation (28) is valid under nominal tolerance conditions and assuming perfectly differential in-series connection structures. The *p*-fold symmetry of the sensor design then leads to the same induced voltage in every winding—but phase-shifted by $\delta_{p}$ electrically for the main harmonic. In typical sensor designs, Equation (28) is sufficiently accurate for all practical purposes.

Here, we take some of the naming conventions from [37]. $A_{p}$ is the main *p*-th-order harmonic of the induced signal in a single winding of one coil. For most analyses, it is sufficient to focus on a single coil. In the numerical results, we include the signal $B_{p}$ of the second coil. Under the previous assumptions, the signal in second coil $B_{p}$ is essentially the same, but phase-shifted by pθ (Equation (25)). Higher harmonic orders are denoted by appropriate subscript.

Note that the amplitude only *increases* by constructive interference compared to the voltage of a single winding up to

(29) $$\delta_{p}<\frac{2\pi }{n_{w}+1}\;,$$

above which $\Sigma A_{p}$ *decreases* compared to a single winding, up to the first zero at complete destructive interference at

(30) $$\delta_{p}=\frac{2\pi }{n_{w}}\;.$$

Note that additional regions of constructive interference may be present at even larger phase shifts.

The resulting effective phase of the signal $\Sigma A_{p}$ is simply shifted to the phase of the winding in the middle if $n_{w}$ is odd, or to the center of the two middle windings if $n_{w}$ is even. This is also evident from Figure 6.

### 2.5. Effect on the Angular Error

As discussed in detail in [37], a systematic *angular error* is caused by disturbance harmonics in the sine and cosine signals of the sensor. The arctangent operation then transforms these harmonics in the signals into an effective angular error. By utilizing the fact that the phase shift $\delta_{p}$ affects the harmonics in the induced voltage signals differently based on their harmonic order, the angular error can effectively be decreased by enforcing destructive interference of higher-order harmonics while keeping constructive interference of the main harmonic. This in turn decreases the harmonic distortion, which leads to a lower angular error compared to a single-winding design.

In a typical inductive rotary position sensor design, odd harmonic orders dominate the harmonic distortion, since even orders are intrinsically eliminated by the differential coil design. Further, higher orders typically decay rapidly in amplitude, so the third harmonic distortion is most dominant. Therefore, $A_{3p}\gg A_{n}$ for n∈N∖{p,3p} and specifically, $A_{3p}\gg A_{5p}$. Since the amplitude of higher-order harmonics affects the angular error relative to the amplitude of the main harmonic, we normalize to $A_{p}$ to obtain a measure for the angular error.

Note that reduction in the angular error by utilizing multiple windings is mostly relevant for two-phase designs, since three-phase designs exhibit intrinsic third harmonic cancellation in the Clarke transform [34]. We therefore focus mainly on the two-phase case. However, three-phase designs can benefit from a reduced angular error as well by additional fifth harmonic suppression (Table 1).

**Figure 7:**
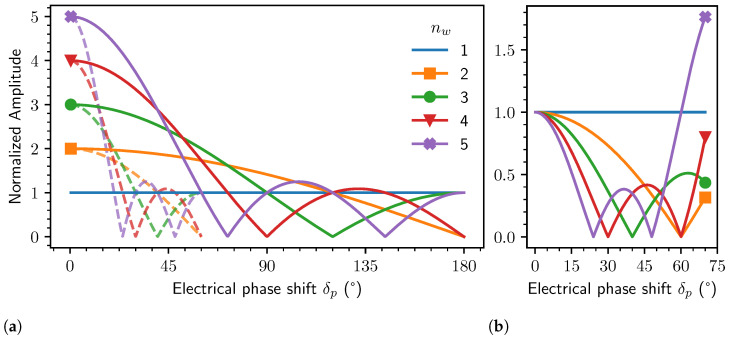
Normalized amplitude of the effective induced voltage $V_{\mathrm{RX}}$ main harmonic of order *p* (solid lines) and 3p (dashed) given by Equation (28) for $n_{w}$ in-series windings dependent on the *electrical* phase shift $\delta_{p}$ between windings (**a**). Amplitudes are normalized to the induced voltage received by a single winding ($n_{w}=1$). The ratio of the harmonic 3p normalized to the amplitude of the main harmonic *p* of the induced voltage (**b**) is a metric for the resulting angular error, which initially reduces with increasing phase shift $\delta_{p}$.

A decrease in the angular error by suppression of the third harmonic (Figure 7b) can be expected in the range

(31) $$\begin{array}{cc} 0<\delta_{p}<\frac{2\pi }{n_{w}+1}\; & \mathrm{for}\;n_{w}>2\;, \end{array}$$

(32) $$\begin{array}{cc} 0<\delta_{p}<\frac{\pi }{2}\; & \mathrm{for}\;n_{w}=2\;. \end{array}$$

In this range, the third harmonic distortion ratio of the effective in-series signal is lower, compared to a single winding, so that:

(33) $$\frac{A_{p}}{A_{3p}}\frac{\Sigma A_{3p}}{\Sigma A_{p}}<1$$

which is the ratio of the normalized distortion of the sum signal and normalized distortion of a single winding, written compactly.

There are several possible phase shifts between windings which result in complete destructive interference of the third harmonic. The first optimum at smallest $\delta_{p}$ is located at

(34) $$\delta_{p}=\frac{2\pi }{3n_{w}}\;.$$

Analogous to Equation (34), a complete destructive interference of the fifth harmonic occurs at

(35) $$\delta_{p}=\frac{2\pi }{5n_{w}}\;.$$

Maximum constructive interference of the third harmonic and, therefore, high resulting angular error occur at $\delta_{p}=\frac{2\pi }{3}$. However, this range is never reached with uniform spacing of windings (Equation (26), Table 1). In fact, by utilizing Equation (26), the phase shift between windings is always smaller than the critical shift from Equation (34), which guarantees a reduction in the third harmonic in any case for two-phase designs.

In case of uniformly spaced windings, the harmonic suppression ratio quickly converges with increasing number of windings:

(36) $$\begin{array}{cc} \lim_{n_{w}\to \infty }\frac{A_{p}}{A_{3p}}\frac{\Sigma A_{3p}}{\Sigma A_{p}}\; & \underset{m=2}{=}\;\frac{1}{3} \end{array}$$

(37) $$\begin{array}{cc} \lim_{n_{w}\to \infty }\frac{A_{p}}{A_{5p}}\frac{\Sigma A_{5p}}{\Sigma A_{p}}\; & \underset{m=3}{=}\;\frac{1}{5} \end{array}$$

for the two-phase and three-phase cases, respectively (Table 1).

Higher induced voltages can be achieved by minimizing the winding phase shift up until the fabrication limit imposed by the PCB design rules. Lower angular errors in two-phase designs can typically be achieved by increasing the phase shift as close as possible to Equation (34). In three-phase designs, choosing the winding phase shift as close as possible to Equation (35) is likely close to optimal.

### 2.6. Rules for In-Series Connection of Windings

In Section 2.3, the fundamental connection structure (Figure 4) for the electrical serial connection is presented. However, it is non-trivial to determine *where* these connection structures must be placed in a specific layout in order to obtain a valid design. If this is not done carefully, windings might be electrically shorted, connected with wrong polarity, or even connected to the wrong coil. The connection pattern for a valid in-series connection is highly dependent on the design parameters of the system and must be determined specifically based on *p*, *m*, and $n_{w}$. Note that this could be carried out *by hand* in a trial-and-error fashion, but this is tedious and prone to errors, especially in designs with a large number of windings. Instead, we present a closed-form solution for placement of the connections that is generally applicable to any design for which a solution exists.

In the following, a *connection* refers to the entire differential connection structure, consisting of the two trace segments on their respective layers as a pair. By following the process outlined in Section 2.2, one obtains $2n_{w}$ shorted loops for each of the *m* coils. The goal of the connection algorithm is to replace these short circuits with in-series connections to obtain a single shorted loop for each coil, which is then finally connected to the demodulating IC—and then no longer shorted. For this reason,

(38) $$n_{c}=2n_{w}-1$$

connections have to be placed in a coil. Note that the previously introduced *reversal points* also count towards the number of connections. In total, an odd number of connections is placed per coil. Solutions with more than $n_{c}$ connections might be possible, but have no practical value.

A solution for serial connection of multiple coil windings, utilizing connections on either *only the inner radius* or *only the outer radius* of the coil system, always exists if

(39) p>m,

i.e., if the periodicity of the design is larger than the number of phases. Note that Equation (39) particularly does not depend on the number of windings $n_{w}$. The number of reversal points $n_{r}$ per individual coil must not exceed the number of windings per coil; therefore, $n_{r}\leq n_{w}$. When utilizing $n_{r}=n_{w}$ reversal points in the design, Equation (39) is not a tight bound. A solution for connection exists if

(40) 2p>m,

which is the tight upper bound for Equation (39) in the case of $n_{r}=n_{w}$.

#### General Solution

A valid *pattern* for in-series connection of windings applicable to any combination of design parameters (*p*, *m*, $n_{w}$) fulfilling Equations (39) or (40) can be derived in closed form. This connection style is further referred to as the *general solution*. We restrict the patterns to the two common cases: no reversal points ($n_{r}=0$) or one reversal point for each winding ($n_{r}=n_{w}$).

As a prerequisite, connections between windings are described in a structured manner in an adjacency matrix, further referred to as the *connection matrix*. Connections are not directional since they represent only an electrical path between two points of the coil system. Therefore, only the lower triangular part of the connection matrix is utilized. The matrix has a square shape; indices represent the via positions of all coils and windings in the order that they occur with increasing angle φ.

We assume 0-based indexing in the following. At the positions where two vias are replaced with a connection segment, the corresponding value in the matrix is set to 1, identified with the row/column index of the original vias. The remaining non-connected vias still in place are set to 0.

**Figure 8:**
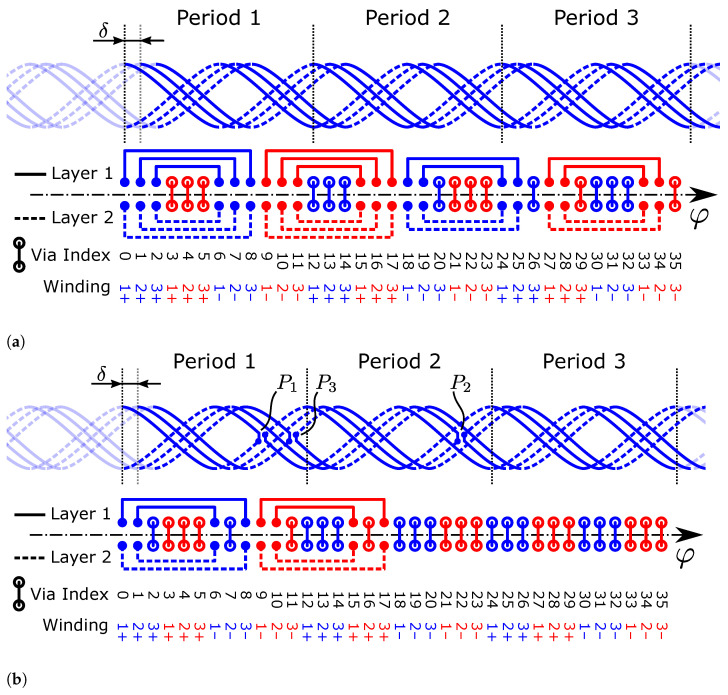
Connection layout of the general solution for in-series connection of three windings ($n_{w}=3$) of a two-phase design (m=2, blue and red). It is applicable to any periodicity p≥3 with *no reversal points* $n_{r}=0$ (**a**) or p≥2 with *one reversal point per winding* (**b**) of the coil system ($n_{r}=n_{w}=3$). Connections are placed symmetrically on both layers, leading to a differential structure. A connection completely replaces the via at its start and end location, so there is no connection between layers at these points. If more periods are available, the connections could be spread out in order to reduce the nesting level from two to one connection. The half-period in which to place the reversal points ($P_{1}$, $P_{2}$, $P_{3}$) can be chosen arbitrarily. The receiver coil centerlines and reversal points are shown only for the first coil (blue) for clarity.

An example connection matrix is given by

(41) $$C=\left[\begin{array}{cccccccccc} 0 & \; & & \; & & \; & & \; & & \\ 0 & 0 & \; & & \; & & \; & & \; & \\ 0 & 0 & 0 & \; & & \; & & \; & & \\ 0 & 0 & 0 & 0 & \; & & \; & & \; & \\ 0 & 0 & 0 & 0 & 0 & \; & & \; & & \\ 0 & 0 & 0 & 0 & 0 & 0 & \; & & \; & \\ 0 & 0 & 1 & 0 & 0 & 0 & 0 & \; & & \\ 0 & 1 & 0 & 0 & 0 & 0 & 0 & 0 & \; & \\ 1 & 0 & 0 & 0 & 0 & 0 & 0 & 0 & 0 & \cdots \\ \vdots & \vdots & \vdots & \vdots & \vdots & \vdots & \vdots & \vdots & \vdots & \ddots \end{array}\right]$$

which represents a subset of the connections in Figure 8. Specifically, the first group of three nested connections is present in the range shown in Equation (41).

Without reversal points, i.e., $n_{r}=0$, the connection matrix for the general solution is obtained by

(42) $$C_{i,j}=\left\{\;\begin{array}{ccc} 1 & \mathrm{if}\; & j=\delta_{y}+k\;\wedge \;i=\delta_{y}+\left(m+1\right)n_{w}-1-k\\ \; & \; & \forall k\in \{0,1,\ldots ,n_{w}-1\}\;,\\ 1 & \mathrm{if}\; & j=\delta_{y}+\delta_{2}+k\;\wedge \;i=\delta_{y}+\delta_{2}+\left(m+1\right)n_{w}-2-k\\ \; & \; & \forall k\in \{0,1,\ldots ,n_{w}-2\}\;,\\ 0 & \mathrm{else}\;, \end{array}\right.$$

for all $y\in \{0,1,\ldots ,n_{w}-1\}$, where $\delta_{y}=y\left(m+1\right)n_{w}$ and $\delta_{2}=m\left(m+1\right)n_{w}$.

If one reversal point is utilized per winding, i.e., $n_{r}=n_{w}$, the general solution is given by

(43) $$C_{i,j}=\left\{\;\begin{array}{ccc} 1 & \mathrm{if}\; & k\leq \frac{1}{2}n_{w}-1\;\wedge \;j=\delta_{y}+k\;\wedge \;i=\delta_{y}+\left(m+1\right)n_{w}-1-k\\ \; & \; & \forall k\in \{0,1,\ldots ,n_{w}-2\}\;,\\ 1 & \mathrm{if}\; & k>\frac{1}{2}n_{w}-1\;\wedge \;j=\delta_{y}+k\;\wedge \;i=\delta_{y}+\left(m+1\right)n_{w}-2-k\\ \; & \; & \forall k\in \{0,1,\ldots ,n_{w}-2\}\;,\\ 0 & \mathrm{else}\;, \end{array}\right.$$

for all $y\in \{0,1,\ldots ,n_{w}-1\}$ where $\delta_{y}=y\left(m+1\right)n_{w}$.

**Figure 9:**
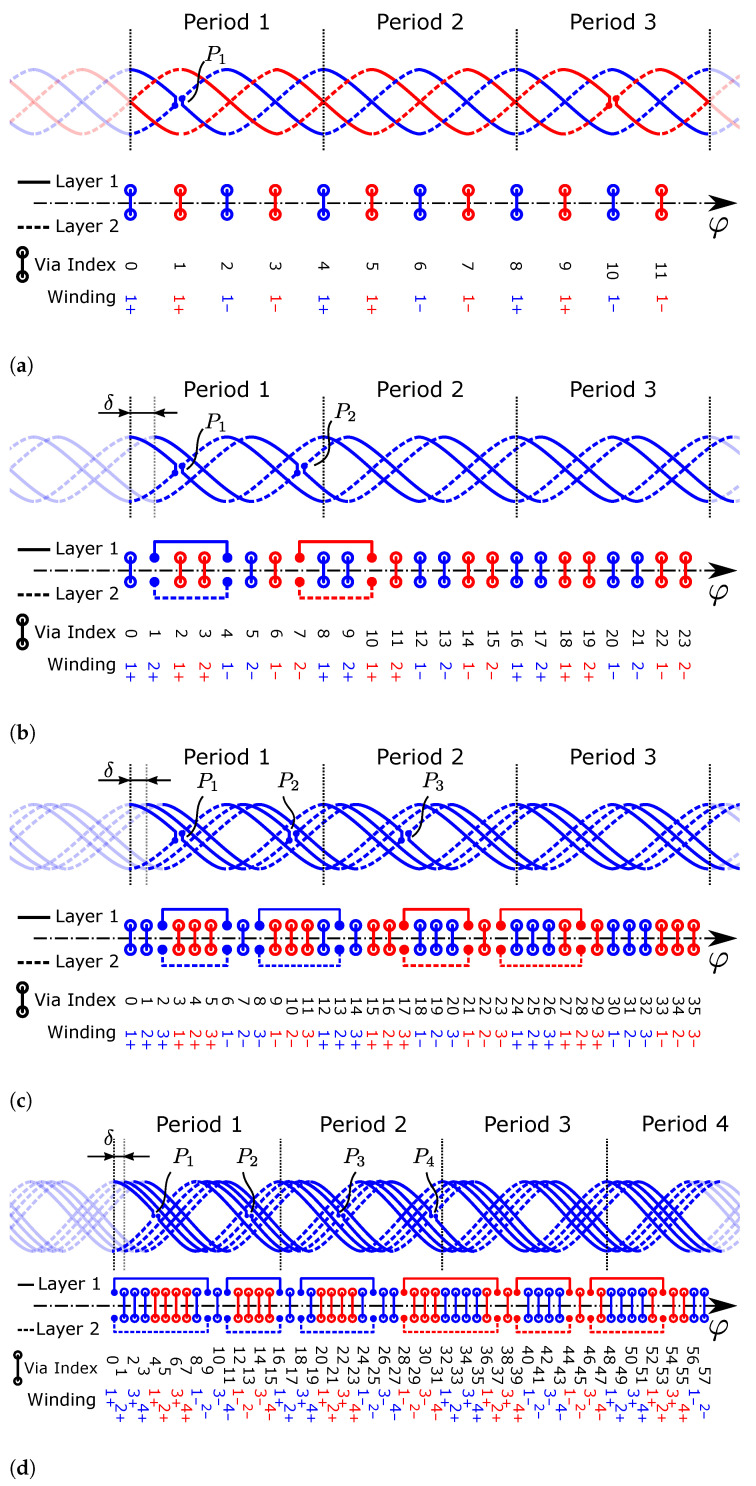
Connection structures used in the sensor application example coil designs. For clarity, receiver coil centerlines are only shown for one phase (blue) of the coil system for $n_{w}>1$. In all designs, one reversal point ($P_{1},P_{2},\ldots$) is used in each winding. (**a**) $n_{w}=1$, $n_{r}=1$. (**b**) $n_{w}=2$, $n_{r}=2$. (**c**) $n_{w}=3$, $n_{r}=3$. (**d**) $n_{w}=4$, $n_{r}=4$.

The connection layout obtained by utilizing the general solution is depicted in Figure 8a for a design without reversal points and in Figure 8b for a design including one reversal point for each winding. Connection patterns differing from the general solution are possible and can be advantageous; however, they cannot be easily described in closed form. Some examples utilizing the minimum number of required nested connections are given in Figure 9. The connection variant without reversal points is particularly useful in dense designs, where the reversal point vias would lead to intersections or design rule violations.

Connections between windings cost *radial space* and, therefore, reduce the effective area of the receiver coil by a small amount if the same mechanical constraints remain. In order to maximize the effective area and therefore the induced signal amplitude in the receiver coil, it is beneficial to minimize the number of nested radial layers of the series connections. We provide *optimal* solution examples with regard to the number of nested connections for $n_{w}=\left\{2,3,4\right\}$ in Figure 9.

The maximum number of radially nested connections of the general solution is equal to $n_{w}$ when not utilizing reversal points (Equation (42)) and $n_{w}-1$ when utilizing one reversal point per winding (Equation (43)). This does not depend on the periodicity or number of coils in the design.

## 3. Finite Element Simulation of Design Examples

**Figure 10:**
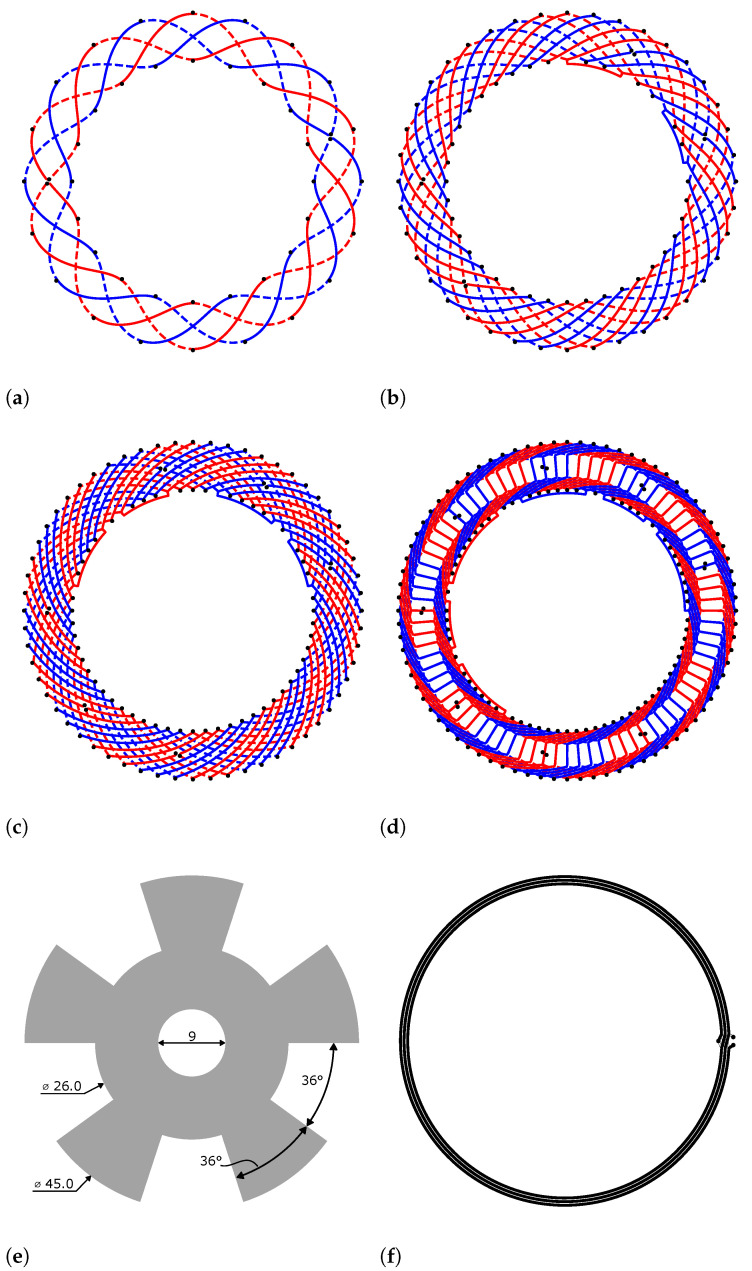
Selected example receiver coil layouts (**a**–**d**), target (**e**), and transmitter coil (**f**). The corresponding phase (parameter *m*) of the receiver coils is indicated by color (cosine blue, sine red). PCB layers are indicated by linestyle. Top layer (closer to target) with solid lines; bottom layer with dashed lines. Dimensions are given in millimeters. (**a**) $n_{w}=1$, cosine function, ϵ=0. (**b**) $n_{w}=2$, modified cosine, ϵ=0.2. (**c**) $n_{w}=3$, triangle, ϵ=1. (**d**) $n_{w}=4$, modified rectangle. (**e**) Target, thickness 1 mm. (**f**) TX coil, 3 windings on 2 layers.

In order to investigate the effect of the discussed parameters, we created coil designs for all combinations of $n_{w}\in \left\{1,2,3,4\right\}$ and receiver coil centerline functions cosine, ϵ=0.1, ϵ=0.2, triangle and modified rectangle that are possible without geometric intersections. In total, this leads to 18 coil layouts, as evident from Table 2. Excluded due to intersections are cosine and modified-cosine (ϵ=0.1) centerline functions for the case $n_{w}=4$. Representative coil layouts are shown in Figure 10. The corresponding in-series connection structures for each number of windings are detailed in Figure 9.

The presented example (Figure 10) is typical for rotary position sensing for motor control applications. The periodicity p=5 is well suited, e.g., for control of a motor with 10 poles. The total diameter of the coil system including the transmitter coil is approximately 46 mm; the target diameter is 45 mm. While it is possible to also construct smaller designs, the presented example is well suited for a wide range of applications and motor sizes. A detailed list of parameters to reproduce the design can be found in Table A1.

Finite element simulations are performed with the *Ansys Maxwell 3D* frequency domain solver with harmonic excitation at f=4 MHz. The bounding box offset is equal to 40 mm in every direction from the sensor geometry and includes a radiation boundary condition. The final mesh consists of approximately 450,000 elements for the smallest model and 1,500,000 elements for the largest model. Rotation of the target is performed in discrete 1° steps, covering the full range of 0–359° for each design. On the target surface, an impedance boundary condition is utilized.

The signals induced in the receiver coils are decomposed into their harmonics by discrete Fourier transform of a full mechanical rotation for detailed analysis (Table 3). The increase in the induced voltage by utilizing multiple windings is evaluated by normalization to the amplitude induced in the simulation with only a single winding of the respective centerline function, i.e.,

(44) $$\frac{\Sigma A_{p}}{A_{p}}\;.$$

The amplitude gains observed in finite element simulation are in excellent agreement with the increase predicted by the analytical calculation in Equation (28). This applies to all tested centerline functions. The maximum deviation is 0.5%.

The third *electrical* harmonic, i.e., the harmonic of order 3p, dominates the distortion of the voltage signals. All other harmonics are approximately one order of magnitude smaller—with some exceptions in case of the rectangular centerline function, where the fifth electrical harmonic is also significant. Since the effect on the angular error is determined by the *ratio* to the main harmonic of order *p*, the suppression ratio is analyzed. In order to compare the reduction with the expected value from theory, the suppression ratio for a specific number of windings $n_{w}$ is normalized to the harmonic of a single winding of the respective centerline function at unit amplitude of the main harmonic, leading to:

(45) $$\frac{A_{p}}{A_{3p}}\frac{\Sigma A_{3p}}{\Sigma A_{p}}\;.$$

The agreement between simulation results and expected suppression ratio is very good for all centerline functions except the pure cosine function. The relative error is smaller than 8%, but in the case of the cosine function, it is up to 20%. However, it is important to note that the amplitude of the third harmonic is already very small in this case—for $n_{w}=1$, the distortion ratio $\frac{\Sigma A_{3p}}{\Sigma A_{p}}\approx 0.1\%$. In order to obtain the suppression ratio, the distortion ratio for, e.g., $n_{w}=3$ of $\frac{\Sigma A_{3p}}{\Sigma A_{p}}\approx 0.05\%$ is normalized to the result of the $n_{w}=1$ simulation by calculating the ratio of the two values. Therefore, inaccuracies in the finite element simulation are amplified significantly in the calculation of this ratio in case of small harmonic distortion. The error difference between the channels *A* and *B* is also attributed to imperfect FEM simulation. Other centerline functions exhibit higher intrinsic harmonic distortion relative to the main harmonic; therefore, it is much more easily resolved in simulation and the error values are smaller.

As expected based on the results of the analysis in Section 2.1.1 and Section 2.5, the lowest angular error in our example is achieved with a pure sinusoidal centerline function and three equidistantly spaced windings ($n_{w}=3$), i.e., the largest number of windings possible with this centerline function. The error is reduced by 65% compared to the conventional design with just one winding, while the amplitude is increased by 174%. Even smaller angular error would be expected with $n_{w}=4$. However, a design with four windings and pure cosine centerline function is not possible without intersections of the trace geometry for the selected sensor parameters.

Utilizing a rectangular centerline function results in an amplitude merely 4% larger than the cosine variant, but results in an angular error which is more than 10 times higher. A rectangular function with as many windings as possible might therefore be considered in an application where the amplitude is critical and the angular error of the sensor is only a secondary consideration.

Receiver coil designs utilizing a pure triangular centerline function are rarely useful, since this choice leads to excessive angular error at a lower induced voltage compared to a fine-tuned modified cosine centerline function. A rectangular centerline function maximizes the induced voltage, at the expense of high angular error (one order of magnitude in our examples).

Out of the analyzed centerline functions, the pure cosine generally provides lowest angular error at good induced voltage amplitudes, provided that the resulting layout with the desired number of windings does not violate PCB design rules. In such a case, switching to a modified cosine centerline and increasing ϵ until design rule compliance will provide the best trade-off.

At first glance, it might seem unintuitive or even incorrect that the design with a rectangle centerline function exhibits lower angular error for $n_{w}=1$ compared to the triangle centerline function, even though the *amplitudes* of both the third and the fifth harmonics in the voltage signals are higher for the rectangle variant. However, the superposition of the resulting angular error harmonics leads to partial cancellation of the fourth harmonic of the angular error in the case of the rectangle centerline function. With increasing number of windings, the voltage signal harmonics are subject to different levels of suppression, and their phase changes as well; therefore, the effect is not pronounced for $n_{w}>1$.

**Figure 11:**
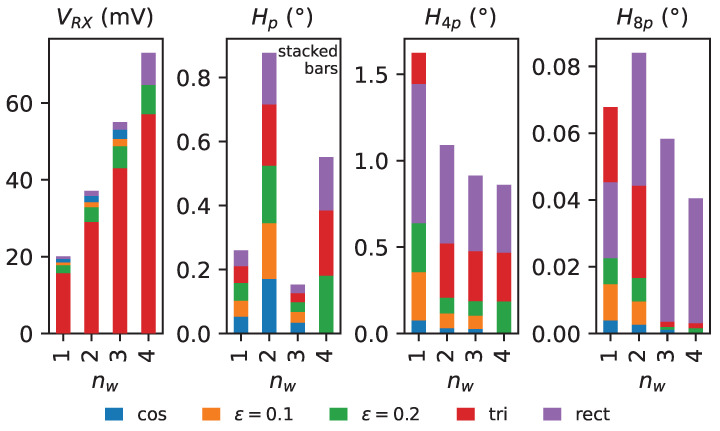
Comparison of induced voltage amplitudes in the receiver coils and selected angular error harmonics $H_{n}$ for our example design with various centerline function types $r_{c}\left(\tilde{\varphi }\right)$ and different number of windings $n_{w}$. Numerical data are available in Table 2. Note that, *except for $H_{p}$*, bars are not stacked but overlaid; the absolute value on the y-axis is then directly representative for any centerline function. Angular error is given in *electrical* degrees.

The top three dominant harmonics of the angular error with largest amplitudes are shown in Figure 11. The angular error harmonic of order *p* does not significantly depend on the choice of centerline function. Therefore, the corresponding bars in Figure 11 are stacked on top of each other for better readability. Rather, the choice of connection pattern for the multiple windings causes a small additional surface area of the receiver coil in the *z*-direction of the PCB, due to the non-zero spacing between layers. This in turn leads to an increased direct coupling of the TX coil field into the receiver coils, which is apparent as an offset, i.e., the zeroth-order harmonic of the voltage signals (Table A2). As discussed in [37], voltage offsets leads to an angular error harmonic of order *p*. This effect is more pronounced for an even number of windings, since the cancellation effect of the surface area spanned in the *z*-direction with varying inverted surface-normal orientation in the connection structures has to be considered. Even though the total absolute surface area spanned in, e.g., the $n_{w}=3$ case is larger, the cancellation effect dominates in this case. However, voltage offsets in the coil signals are trivially compensated by the ASIC; therefore, the impact is less relevant in practice.

The harmonic $H_{4p}$ is caused by third and fifth harmonic distortions and significantly depends on the chosen centerline function. As expected, the cosine function exhibits the smallest angular error for all winding numbers where the design is possible. The harmonic suppression effect with increasing number of windings is clearly present for all centerlines. In particular, the modified cosine centerline function with ϵ=0.2 benefits from this effect and increases the chance of acceptance in designs where no other centerline function can fulfill given design rules. The rectangular centerline function performs worst in terms of angular error for all designs except $n_{w}=1$, where the triangle centerline exhibits the highest fourth harmonic of the angular error observed in the examples, with 1.62° electrical error. The previously discussed peculiarity of higher error suppression for the triangle function applies.

The residual harmonic $H_{8p}$ is orders of magnitude smaller than $H_{4p}$ and therefore insignificant for most applications. It is caused by a combination of two effects: higher-order voltage harmonics, such as the seventh order, and the second-order error effect of the third and fifth harmonics. In the case of the cosine centerline function for $n_{w}=3$, the harmonic $H_{8p}$ is even smaller than 0.0005°, which is below the noise floor of the finite element simulation.

The simulation results demonstrate the practicability of the presented methods for real-world applications and highlight the significant benefits obtained from the in-series connection of multiple windings with differential connection structures.

### Limits of the Coil Design Approach

The maximum number of windings which can be fitted in a coil design is mainly constrained by the current limit of PCB manufacturing technology. Positioning tolerances of via holes and copper-to-copper distance contraints due to the etching process lead to design rules which have to be complied with in order to achieve reliable sensor designs. A good overview of the manufacturing process for other sensing applications is given in [38].

By adjusting the ϵ parameter of the modified contour centerline function and by moving vias radially, it is possible to fit more windings given the same constraint—but ultimately, a limit is reached. A possible option is increasing the number of layers of the PCB and duplicating the coil system on those layers, also connected in series, but this introduces additional costs which may be critical in cost-sensitive applications. We exclude such possibilities from our analyses here, since the realization is highly dependent on the specific constraints of the sensor design.

Apart from trace-to-trace and via-to-trace distance limits, the number of vias on the inner radius $r_{i}$ is often a limiting factor in a sensor design—since they are spaced closer together than on the outer radius. Given a via pad diameter of $d_{\mathrm{pad}}$ (potentially also including the design rule distance for spacing between copper surfaces), a rule of thumb for the feasibility of a sensor design is given by

(46) $$2m\;n_{w}\;p\;d_{\mathrm{pad}}<2\pi r_{i}\;.$$

Above this limit, it is not possible to arrange the vias on the same radius. Note that this is not exclusive to designs with multiple windings; it can also be reached with high periodicity *p* or small radius $r_{i}$ at $n_{w}=1$.

In real-world applications, part of the residual angular error is caused by dynamic tolerance effects, such as, e.g., mechanical misalignment under load, which is hard to compensate. This type of error is likely not significantly affected by our proposed method. However, the intrinsic angular error of a coil design due to third harmonic distortion can be greatly reduced by utilizing multiple windings in the coil design.

## 4. Summary and Outlook

In summary, utilizing multiple windings for the receiver coils of inductive position sensors has significant benefits, while no discernible disadvantage could be identified—given that the resulting PCB layout is suitable for fabrication due to PCB design rules. Rather, designs with multiple windings achieve the highest possible degree of effectiveness: *higher* induced voltage amplitude, *lower* harmonics of the angular error, and *unaffected* cost of the design. The method enables designs and opens up the possibility for applications which were previously considered not suitable for the inductive sensing principle, such as large airgaps or very small sensors. Additionally, it often makes harmonic compensation of the angular error of the sensor unnecessary, due to the significantly decreased angular error.

Furthermore, we provided methods and guidelines for how to apply the concept in concrete real-world applications, by, e.g., enabling space-constrained designs by use of modified receiver coil centerline functions. The generally applicable solution for in-series connection of the windings can easily be implemented in an algorithm for automated coil design.

The theoretical considerations with regard to the amplitude gain and reduction in the angular error are validated and confirmed with commercial finite element simulation software, showing excellent agreement between expected values and simulation results.

Future work might include the automated placement of optimized connection structures, while also fulfilling PCB design constraints with regard to copper distances. Further research to improve the optimality of the connection placement compared to the general solution given in closed form could speed up the process of placing connections by hand.

An open topic which we did not cover in the scope of this publication is an in-depth analysis of the influence of the presented approach under tolerance conditions, such as eccentricity of the target. Our expectation is that the angular errors due to such conditions are either unaffected or even reduced, compared to a single winding—though this remains to be proven.

## Figures and Tables

**Table 1:** Phase shift angle, amplitude gain, and harmonic suppression ratios for various numbers of windings $n_{w}$, for *uniform spacing* of the receiver coil windings of two (m=2) and three phases (m=3). In all cases, the amplitude of the induced voltage increases, while the 3rd or 5th harmonic distortion of the induced voltage is reduced, which generally results in lower angular error values. Normalization of $A_{p}=1$ is assumed in the formulas below for calculation of the harmonic suppression ratio.

	m=2			m=3		
$n_{w}$	$\delta_{p}$	$\frac{\Sigma A_{p}}{A_{p}}$	$\frac{A_{p}}{A_{3p}}\frac{\Sigma A_{3p}}{\Sigma A_{p}}$	$\delta_{p}$	$\frac{\Sigma A_{p}}{A_{p}}$	$\frac{A_{p}}{A_{5p}}\frac{\Sigma A_{5p}}{\Sigma A_{p}}$
1	–	1.00	1.00	–	1.00	1.00
2	45.00°	1.85	0.41	30.00°	1.93	0.27
3	30.00°	2.73	0.37	20.00°	2.88	0.23
4	22.50°	3.62	0.35	15.00°	3.83	0.21
5	18.00°	4.52	0.34	12.00°	4.78	0.21
6	15.00°	5.42	0.34	10.00°	5.74	0.21
7	12.86°	6.32	0.34	8.57°	6.69	0.20
8	11.25°	7.21	0.34	7.50°	7.64	0.20
9	10.00°	8.11	0.34	6.67°	8.60	0.20

**Table 2:** Simulation results for various receiver coil geometries of the example designs (Table A1). The receiver coil voltage amplitude is given in millivolts, normalized to a 1V TX excitation amplitude. The mean amplitude gain for $A_{p}$ and $B_{p}$ is calculated w.r.t. to a single winding cosine centerline. Harmonic angular error amplitudes $H_{k}$ of order *k* are given in *electrical* degrees (i.e., the error in mechanical degrees is smaller) and are calculated from uncompensated raw signals. The peak-to-peak angular error $\Delta \phi_{p2p}$ is calculated from offset-compensated signals (subtraction of $A_{0}$, $B_{0}$).

$n_{w}$	$r(\tilde{\varphi })$	$A_{p}$ (mV)	$B_{p}$ (mV)	gain	$\Delta \phi_{p2p}$ **(°)**	$H_{p}$ **(°)**	$H_{4p}$ **(°)**	$H_{8p}$ **(°)**
1	cos	19.35	19.35	0.0%	0.18	0.053	0.075	0.004
	ϵ=0.1	18.44	18.44	−4.7%	0.74	0.050	0.353	0.015
	ϵ=0.2	17.75	17.75	−8.2%	1.33	0.056	0.637	0.022
	tri	15.67	15.67	−19.0%	3.29	0.052	1.624	0.068
	rect	20.08	20.08	3.8%	2.90	0.050	1.443	0.045
2	cos	35.78	35.78	84.9%	0.10	0.171	0.030	0.003
	ϵ=0.1	34.11	34.11	76.3%	0.27	0.174	0.115	0.010
	ϵ=0.2	32.84	32.84	69.7%	0.45	0.179	0.206	0.017
	tri	28.98	28.98	49.7%	1.08	0.192	0.520	0.044
	rect	37.14	37.14	91.9%	2.24	0.161	1.090	0.084
3	cos	53.03	53.03	174.0%	0.07	0.034	0.025	0.001
	ϵ=0.1	50.58	50.58	161.4%	0.22	0.034	0.101	0.001
	ϵ=0.2	48.69	48.70	151.7%	0.40	0.030	0.185	0.002
	tri	42.96	42.96	122.0%	0.96	0.028	0.474	0.003
	rect	55.06	55.06	184.5%	1.85	0.027	0.913	0.058
4	ϵ=0.2	64.67	64.68	234.2%	0.40	0.181	0.184	0.002
	tri	57.04	57.04	194.8%	0.95	0.203	0.466	0.003
	rect	73.08	73.10	277.7%	1.73	0.167	0.861	0.040

**Table 3:** Harmonics in the voltage signals of sine and cosine coils in the example simulations for different centerline functions $r(\tilde{\varphi })$ and number of windings $n_{w}$. Higher-order harmonics are normalized to the main harmonic of order *p*. Amplitude gains and suppression ratios are calculated with regard to a single winding of the respective centerline function. Errors are calculated w.r.t. the expected theoretical values from Table 1.

		Main Harmonic Amplitude Gain				3rd Harmonic × 10^3^		3rd Harmonic Suppression Ratio				5th Harmonic × 10^3^	
$n_{w}$	$r(\tilde{\varphi })$	$\frac{\Sigma A_{p}}{A_{p}}$	**Error**	$\frac{\Sigma B_{p}}{B_{p}}$	**Error**	$\frac{\Sigma A_{3p}}{\Sigma A_{p}}$	$\frac{\Sigma B_{3p}}{\Sigma B_{p}}$	$\frac{\frac{\Sigma A_{3p}}{\Sigma A_{p}}}{\frac{A_{3p}}{A_{p}}}$	**Error**	$\frac{\frac{\Sigma B_{3p}}{\Sigma B_{p}}}{\frac{B_{3p}}{B_{p}}}$	**Error**	$\frac{\Sigma A_{5p}}{\Sigma A_{p}}$	$\frac{\Sigma B_{5p}}{\Sigma B_{p}}$
1	cos	1.00	0.0%	1.00	0.0%	1.15	1.21	1.00	0.0%	1.00	0.0%	0.16	0.12
	ϵ=0.1	1.00	0.0%	1.00	0.0%	5.46	5.66	1.00	0.0%	1.00	0.0%	0.65	0.58
	ϵ=0.2	1.00	0.0%	1.00	0.0%	9.82	9.75	1.00	0.0%	1.00	0.0%	1.28	1.40
	tri	1.00	0.0%	1.00	0.0%	25.24	25.10	1.00	0.0%	1.00	0.0%	3.21	3.15
	rect	1.00	0.0%	1.00	0.0%	35.63	35.62	1.00	0.0%	1.00	0.0%	10.44	10.48
2	cos	1.85	0.1%	1.85	0.1%	0.55	0.52	0.48	16.1%	0.43	3.7%	0.06	0.03
	ϵ=0.1	1.85	0.1%	1.85	0.1%	2.16	2.16	0.40	−4.6%	0.38	−7.8%	0.12	0.25
	ϵ=0.2	1.85	0.1%	1.85	0.1%	4.05	4.07	0.41	−0.5%	0.42	0.8%	0.46	0.47
	tri	1.85	0.1%	1.85	0.1%	10.37	10.38	0.41	−0.8%	0.41	−0.1%	1.31	1.26
	rect	1.85	0.1%	1.85	0.1%	14.85	14.77	0.42	0.6%	0.41	0.1%	4.13	4.37
3	cos	2.74	0.3%	2.74	0.3%	0.51	0.48	0.44	20.0%	0.40	8.0%	0.06	0.05
	ϵ=0.1	2.74	0.4%	2.74	0.4%	2.01	1.94	0.37	0.5%	0.34	−6.5%	0.26	0.16
	ϵ=0.2	2.74	0.4%	2.74	0.4%	3.56	3.51	0.36	−1.1%	0.36	−1.6%	0.27	0.33
	tri	2.74	0.4%	2.74	0.4%	9.11	9.12	0.36	−1.3%	0.36	−0.7%	0.84	0.83
	rect	2.74	0.3%	2.74	0.3%	13.10	13.11	0.37	0.5%	0.37	0.5%	2.83	2.84
4	ϵ=0.2	3.64	0.5%	3.64	0.5%	3.45	3.48	0.35	−0.0%	0.36	1.6%	0.26	0.25
	tri	3.64	0.4%	3.64	0.4%	8.87	8.91	0.35	0.1%	0.35	1.0%	0.73	0.78
	rect	3.64	0.4%	3.64	0.4%	12.44	12.58	0.35	−0.6%	0.35	0.6%	2.56	2.46

## Data Availability

Data are contained within the article.

## Abbreviations

The following abbreviations are used in this manuscript:

ASIC	Application-Specific Integrated Circuit
PCB	Printed Circuit Board
TX	Transmitter (coil)
RX	Receiver (coil)
via	Electrical connection between PCB layers
ϕ	Mechanical rotation angle
φ	Angle in cylindrical/polar coordinates
$\tilde{\varphi }$	Periodicity-normalized angle
*r*	Radius
x	Position vector (Cartesian coordinates)
*p*	Sensor periodicity (p-fold symmetry)
*m*	Number of coils (phases)
$n_{w}$	Number of windings per coil
$n_{r}$	Number of reversal points per coil
$A_{k}$	*k*-th harmonic of induced voltage of sine channel
$B_{k}$	*k*-th harmonic of induced voltage of cosine channel
$H_{k}$	*k*-th harmonic of angular error

## Appendix A

**Table A1:** Summary of geometric and electrical parameters of the example design. Unless noted otherwise, distances and sizes are given up to the copper edge, not the centerline. TX centerlines are located at 21.6 mm, 22.1 mm and 22.6 mm radius.

Parameter	Value
Periodicity	p=5
# phases	m=2
# systems	s=1
Target wing angle	36°
Target outer radius	22.5 mm
Target inner radius	13 mm
Target bore diameter	4.5 mm
Target thickness	1 mm
Target material	1.4301 stainless steel
Target conductivity	$\sigma =1.4\;MS\;m^{-1}$
Target rel. permeability	$\mu_{r}=1$
Target airgap	1 mm
PCB layer thickness	0.035 mm
PCB layer distance	0.36 mm
TX trace width	0.25 mm
TX # windings	3
TX # layers	2
TX winding distance	0.25 mm
TX outer radius	22.725 mm
TX frequency	4 MHz
TX excitation	1V (2 $V_{\mathrm{pp}}$)
RX trace width	0.2 mm
RX outer via midpoint radius	20.5 mm
RX inner via midpoint radius	14.65 mm
RX inner radius	14.8 mm
RX outer radius	20.35 mm
RX via hole diameter	0.1 mm
RX via pad diameter	0.3 mm
RX connections radius delta	0.325 mm
RX reversal distance	0.86°
RX modified rectangle $\Delta r_{i}$, $\Delta r_{o}$	0.5045
RX centerline interpolation	0.2 mm

**Table A2:** Raw finite-element simulation results, compressed by selecting relevant harmonics. Coefficients are obtained by discrete Fourier transform of the induced voltage of a full mechanical rotation of the target in 1° steps. Values are normalized to units of μV for all orders except the main harmonic of order p=5 which is given in mV. The DFT is applied to the real part of the voltage, the TX excitation voltage (real) being the phase reference (0°). Continued on the next page.

	$n_{w}=1$					$n_{w}=2$				
	**cos**	ϵ=0.1	ϵ=0.2	**tri**	**rect**	**cos**	ϵ=0.1	ϵ=0.2	**tri**	**rect**
$A_{0}$	+0.43 +0.00i	−0.24 +0.00i	−2.71 +0.00i	−2.77 +0.00i	−2.62 +0.00i	+68.28 +0.00i	+62.36 +0.00i	+67.30 +0.00i	+67.03 +0.00i	+68.84 +0.00i
$B_{0}$	−18.90 +0.00i	−15.26 +0.00i	−19.84 +0.00i	−15.38 +0.00i	−18.96 +0.00i	−89.40 +0.00i	−89.36 +0.00i	−84.67 +0.00i	−78.82 +0.00i	−84.26 +0.00i
$A_{1}$	+0.23 −2.09i	+0.68 −0.47i	−3.31 +0.08i	−1.03 −2.50i	−0.31 −0.27i	−0.54 −0.53i	−2.06 +5.38i	+0.12 −0.21i	−1.24 +0.45i	−7.67 −0.77i
$B_{1}$	−0.43 −0.60i	−4.26 −1.03i	−2.78 −0.29i	−0.48 −1.53i	+0.34 −0.21i	−2.22 −2.46i	−1.39 +4.29i	+0.27 +0.12i	+1.36 −0.07i	−0.75 −1.01i
$A_{2}$	−1.66 +0.30i	−0.98 −0.68i	−3.29 −0.83i	+0.79 −0.73i	−0.37 −0.04i	−0.11 −0.04i	+4.03 +2.70i	−0.70 −0.40i	+0.97 −1.14i	+7.70 +0.83i
$B_{2}$	−0.49 +0.11i	+3.55 +2.94i	−3.04 −0.88i	+1.74 −0.43i	+0.65 −0.48i	+0.67 −2.27i	+3.57 +2.67i	−0.10 +0.05i	−1.07 +0.40i	+1.55 +0.80i
$A_{3}$	+0.65 +1.22i	+0.71 +0.61i	−3.07 −1.25i	+0.99 +2.00i	+0.94 −0.00i	+0.83 −0.38i	+4.80 −1.99i	−0.04 +0.23i	−0.98 +0.90i	−7.19 −0.83i
$B_{3}$	+0.67 +0.75i	−2.92 −3.81i	−3.26 −1.43i	+0.54 +0.53i	−0.64 +0.59i	+2.51 −2.17i	+3.73 −1.94i	−0.04 −0.09i	+1.12 −1.00i	−2.26 −2.63i
$A_{4}$	+0.67 −1.51i	−0.41 −1.04i	−2.94 −1.49i	−2.26 +1.89i	+0.49 +0.02i	+0.26 −0.28i	−0.02 −5.61i	−0.10 +0.01i	+0.27 −1.46i	+7.59 +1.26i
$B_{4}$	+0.45 −0.59i	+0.78 +4.55i	−2.18 −1.72i	−0.45 +1.33i	−0.46 +0.63i	+2.15 +0.25i	−0.38 −4.67i	+0.09 −0.23i	−0.96 +1.34i	+2.38 +2.63i
$A_{5}$	+19.35 −0.13i	+18.44 −0.12i	+17.75 −0.11i	+15.67 −0.09i	+20.08 −0.18i	+32.94 −13.98i	+31.40 −13.31i	+30.24 −12.81i	+26.69 −11.29i	+34.16 −14.58i
$B_{5}$	+0.13 +19.35i	+0.12 +18.44i	+0.11 +17.75i	+0.09 +15.67i	+0.18 +20.08i	+13.98 +32.94i	+13.31 +31.40i	+12.82 +30.24i	+11.30 +26.69i	+14.57 +34.16i
$A_{10}$	+1.27 −1.41i	+1.56 −1.90i	+0.36 −5.41i	−1.87 −2.72i	+0.42 −3.12i	+5.02 −7.99i	+0.63 −9.63i	+4.97 −6.69i	+4.55 −7.82i	+11.25 −2.79i
$B_{10}$	+0.87 +1.91i	−3.45 +0.35i	+0.01 −1.02i	−0.09 +0.34i	+1.03 +2.23i	+4.18 −4.88i	+0.30 −4.59i	+4.39 −1.98i	+5.05 −2.00i	+7.66 −2.44i
$A_{15}$	−2.36 +22.14i	−98.92 +18.51i	−173.94 +12.74i	−395.40 +4.58i	+715.47 +11.32i	+17.98 +8.29i	−16.34 +71.69i	−37.39 +127.64i	−107.82 +280.36i	+215.78 −507.53i
$B_{15}$	+23.40 +1.06i	+16.30 +103.00i	+17.61 +172.17i	+4.25 +393.28i	+10.29 −715.35i	+5.90 −17.65i	+72.96 +10.23i	+128.16 +37.89i	+279.85 +110.36i	−501.53 −221.90i
$A_{20}$	−0.53 +0.71i	−0.47 −1.13i	+3.17 −0.31i	−0.44 −2.37i	−0.53 −0.65i	+0.34 +0.40i	−3.34 −5.13i	−0.23 +0.55i	+0.50 +1.45i	+3.72 +6.39i
$B_{20}$	−0.06 +0.24i	+3.63 +2.36i	+4.42 −1.10i	+0.06 −2.01i	+1.69 −0.29i	+1.60 −1.23i	−2.63 −6.68i	+0.23 −2.64i	−0.66 −3.20i	+1.37 +2.57i
$A_{25}$	+2.07 −2.29i	+11.92 −0.65i	+22.62 +1.06i	+50.32 −0.18i	+209.76 +0.39i	+1.35 −1.66i	+3.14 +2.40i	+7.38 +13.28i	+14.53 +35.09i	+59.59 +141.19i
$B_{25}$	+2.27 −0.45i	+4.55 +9.74i	+3.96 +24.47i	+0.49 +49.31i	−0.59 +210.51i	+0.36 −1.11i	−8.55 −0.32i	−13.77 +6.88i	−33.46 +14.93i	−151.44 +58.21i
$A_{30}$	−1.65 −0.00i	+0.09 +0.64i	−0.31 +2.99i	+0.43 −0.83i	+0.31 +0.50i	−0.70 +0.10i	−0.59 −5.75i	−0.49 +0.26i	−1.08 −1.39i	+0.12 +7.13i
$B_{30}$	−1.00 −0.17i	−2.02 −4.30i	−0.65 +4.54i	+0.92 −1.96i	−0.27 −0.11i	−0.58 +0.59i	+0.22 −4.43i	+0.55 −0.55i	−0.15 +0.59i	−0.91 +0.88i
$A_{35}$	+0.44 +0.35i	−1.86 −0.86i	−7.23 +1.28i	−8.92 −1.01i	+44.20 −0.47i	−0.18 −0.84i	−2.59 −6.95i	−7.01 −4.00i	−15.88 −7.93i	+80.11 +24.88i
$B_{35}$	+0.62 +1.61i	−4.84 +4.69i	−3.51 +6.38i	+0.09 +8.82i	+0.95 −45.53i	+0.90 +0.60i	−0.60 −0.18i	−3.29 +6.68i	−9.27 +16.31i	+34.33 −82.43i
$A_{40}$	−0.52 −0.82i	−0.54 −0.49i	−3.54 +0.18i	+3.21 +0.15i	−0.20 −0.47i	−0.11 −0.37i	+2.90 −4.21i	−0.34 +0.15i	−0.72 +1.22i	−4.13 +6.40i
$B_{40}$	−0.50 −0.62i	+0.35 +4.85i	−2.34 −0.76i	+0.97 −0.90i	+0.29 +0.49i	−0.54 −0.86i	+1.86 −3.90i	−0.13 +0.37i	+0.65 −1.18i	+0.00 +3.69i
	$n_{w}=3$					$n_{w}=4$				
	**cos**	ϵ=0.1	ϵ=0.2	**tri**	**rect**	ϵ=0.2	**tri**	**rect**		
$A_{0}$	+24.05 +0.00i	+26.08 +0.00i	+22.18 +0.00i	+18.66 +0.00i	+21.57 +0.00i	+125.46 +0.00i	+127.92 +0.00i	+126.94 +0.00i		
$B_{0}$	−26.39 +0.00i	−18.05 +0.00i	−16.48 +0.00i	−13.02 +0.00i	−17.16 +0.00i	−159.12 +0.00i	−153.44 +0.00i	−165.69 +0.00i		
$A_{1}$	+0.63 −1.76i	+2.71 +4.40i	+3.17 −2.61i	+0.30 −0.61i	−0.66 −2.67i	−0.20 +0.34i	+0.12 +0.49i	+2.16 +8.67i		
$B_{1}$	+0.61 +0.14i	−0.75 −2.65i	+1.78 −1.57i	−0.99 +1.02i	+1.59 +1.37i	−0.18 +0.27i	+2.32 −0.21i	−0.33 +0.42i		
$A_{2}$	−1.52 −0.85i	+2.33 +0.53i	−0.61 +3.67i	+0.06 +1.07i	+1.77 −1.15i	+0.35 −0.14i	−0.98 +0.52i	+2.10 −4.53i		
$B_{2}$	−1.12 −0.32i	−2.52 −0.38i	−0.60 +2.19i	−0.14 −1.40i	+0.05 +3.22i	+0.54 −0.52i	+0.59 −1.29i	+0.34 −1.06i		
$A_{3}$	−0.92 +1.65i	+1.47 +1.87i	−1.37 −3.84i	−0.63 −0.28i	+1.32 +0.54i	−0.01 −0.41i	+0.06 −0.47i	+3.16 +0.14i		
$B_{3}$	+0.72 +0.81i	+0.01 +1.63i	−0.50 −2.22i	+1.33 +0.63i	−2.17 +1.38i	−0.20 −0.31i	−0.03 −2.79i	+1.90 −0.14i		
$A_{4}$	+0.86 +1.26i	−1.85 −1.31i	+3.77 +1.88i	+0.90 −0.51i	+0.46 +1.38i	−0.49 +0.23i	+0.09 −0.32i	−0.24 −11.23i		
$B_{4}$	−0.42 −0.71i	+2.08 −0.45i	+1.58 +1.20i	−1.44 +0.47i	−2.13 −1.21i	+0.06 +0.63i	+0.48 −3.00i	−0.09 −1.10i		
$A_{5}$	+45.68 −26.93i	+43.58 −25.67i	+41.96 −24.71i	+37.02 −21.79i	+47.38 −28.05i	+53.44 −36.41i	+47.15 −32.10i	+60.30 −41.29i		
$B_{5}$	+26.94 +45.68i	+25.68 +43.58i	+24.72 +41.96i	+21.79 +37.02i	+28.06 +47.37i	+36.42 +53.45i	+32.11 +47.15i	+41.30 +60.31i		
$A_{10}$	−1.44 −2.36i	−3.48 +0.49i	−7.18 +0.62i	−2.29 +0.25i	−3.49 +1.12i	−3.03 +0.07i	−4.10 +0.43i	−12.55 +11.90i		
$B_{10}$	+4.93 −2.93i	+4.42 −1.16i	+4.25 −1.88i	+3.19 −2.70i	+4.32 −1.62i	−2.48 +6.13i	−2.96 +8.98i	−0.74 +7.47i		
$A_{15}$	+26.43 −4.43i	+23.92 +98.63i	+14.21 +172.61i	+9.49 +391.38i	+16.30 −721.11i	+68.24 +212.29i	+111.63 +493.53i	−161.19 −894.59i		
$B_{15}$	−3.09 −25.18i	+95.64 −20.81i	+169.96 −18.19i	+391.78 −6.71i	−721.54 −15.99i	+214.60 −67.13i	+496.06 −109.29i	−905.69 +160.31i		
$A_{20}$	+1.02 −4.58i	+2.06 +0.80i	−3.26 −0.46i	+0.74 −3.47i	+2.84 −3.44i	+0.73 −2.59i	+1.60 −1.40i	+3.03 +10.69i		
$B_{20}$	+2.85 −1.93i	+3.29 −3.26i	−0.23 −1.80i	+3.15 −0.50i	−0.84 −3.30i	−0.29 +0.75i	−0.53 −0.38i	−0.39 +2.88i		
$A_{25}$	+0.12 −3.09i	+13.06 +1.65i	+10.28 +8.37i	+31.17 +18.38i	+135.41 +76.65i	+16.84 +2.55i	+40.98 +8.07i	+181.62 +44.59i		
$B_{25}$	+2.46 +0.21i	−5.14 +6.55i	−8.07 +14.02i	−17.27 +31.37i	−76.50 +136.63i	−1.90 +15.87i	−7.99 +43.68i	−34.41 +176.83i		
$A_{30}$	−1.36 −1.02i	+3.70 −0.91i	−3.84 +1.95i	+0.80 +0.56i	−0.43 −2.59i	−1.47 −0.16i	−1.21 +0.43i	+10.41 +7.20i		
$B_{30}$	+0.39 −0.03i	−1.14 +0.98i	−1.92 +0.33i	−0.27 −1.46i	+1.48 −2.25i	−0.95 −0.56i	+0.90 +0.68i	+0.65 +1.18i		
$A_{35}$	−0.68 +0.34i	−1.34 −4.69i	−6.31 +3.65i	−5.71 +2.99i	+27.70 −18.01i	−1.91 +2.45i	−3.20 +6.09i	+30.61 −26.89i		
$B_{35}$	+0.07 −0.75i	+3.12 +3.23i	−0.43 +4.32i	+1.75 +6.38i	−17.68 −27.83i	+2.53 +2.57i	+4.10 +2.70i	−25.91 −18.14i		
$A_{40}$	−0.77 −0.14i	+0.44 −2.88i	−3.41 +2.57i	−0.36 −0.82i	−2.46 +0.51i	+0.42 −1.17i	−0.16 −0.01i	+12.96 −4.10i		
$B_{40}$	−0.51 +0.03i	+0.11 −1.69i	−2.04 +1.61i	+0.66 +1.29i	+1.99 +1.10i	+0.30 +0.26i	−1.03 +1.81i	+2.29 −0.56i		
